# Naphthoquinone as a New Chemical Scaffold for Leishmanicidal Inhibitors of *Leishmania* GSK-3

**DOI:** 10.3390/biomedicines10051136

**Published:** 2022-05-14

**Authors:** Victor Sebastián-Pérez, Paula Martínez de Iturrate, Montserrat Nácher-Vázquez, Luis Nóvoa, Concepción Pérez, Nuria E. Campillo, Carmen Gil, Luis Rivas

**Affiliations:** 1 Centro de Investigaciones Biológicas Margarita Salas (CIB-CSIC), 28040 Madrid, Spain; victorsebastianperez@gmail.com (V.S.-P.); pmisanz@cib.csic.es (P.M.d.I.); montserratnv@gmail.com (M.N.-V.); novoarodriguezluis@gmail.com (L.N.); nuria.campillo@csic.es (N.E.C.); 2 Instituto de Química Médica (IQM-CSIC), 28006 Madrid, Spain; iqmm305@iqm.csic.es

**Keywords:** leishmanicidal drugs, ligand-based drug discovery, hit optimization, leishmaniasis, GSK-3

## Abstract

More than 1 billion people live in areas endemic for leishmaniasis, which is a relevant threat for public health worldwide. Due to the inadequate treatments, there is an urgent need to develop novel alternative drugs and to validate new targets to fight this disease. One appealing approach is the selective inhibition of protein kinases (PKs), enzymes involved in a wide range of processes along the life cycle of *Leishmania.* Several PKs, including glycogen synthase kinase 3 (GSK-3), have been validated as essential for this parasite by genetic or pharmacological methods. Recently, novel chemical scaffolds have been uncovered as *Leishmania* GSK-3 inhibitors with antiparasitic activity. In order to find new inhibitors of this enzyme, a virtual screening of our in-house chemical library was carried out on the structure of the *Leishmania* GSK-3. The virtual hits identified were experimentally assayed both for leishmanicidal activity and for in vitro inhibition of the enzyme. The best hits have a quinone scaffold. Their optimization through a medicinal chemistry approach led to a set of new compounds, provided a frame to establish biochemical and antiparasitic structure–activity relationships, and delivered molecules with an improved selectivity index. Altogether, this study paves the way for a systemic search of this class of inhibitors for further development as potential leishmanicidal drugs.

## 1. Introduction

More than 20 protozoan species of the genus *Leishmania* are responsible for the variety of clinical syndromes associated with human leishmaniasis. This heterogeneous pathology is grouped into three major clinical forms: cutaneous (in many cases self-healing), mucocutaneous, and visceral, that evolves fatally unless treated [1]. The incidence of leishmaniasis is estimated between 700,000 and 1 million new cases with 7000–15,000 annual deaths, and more than 1 billion people living in endemic areas. Its distribution encompasses a wide variety of ecological systems, mostly in tropical and subtropical areas, with an expanding distribution towards higher latitudes, driven by climate change and human displacement or migrations from or into endemic areas [2].

Chemotherapy is the current standard method to fight the disease, and is in turn limited to five drugs with important drawbacks, such as rising resistance, high cost, or undesirable side effects [3,4]. To soothe the health impact of this problem, combination therapy [5] and drug repurposing [6] are the most suitable alternatives for short- and medium-term treatment, respectively. Therefore, there is an urgent need to develop novel alternatives in terms of more effective treatments to fight the disease and to validate new targets.

Protein kinases (PKs) are one of the largest gene families in the genome of eukaryotes where they play a critical role in signal transduction and other cellular processes. They have been involved in cancer, diabetes, neurodegenerative, cardiovascular, developmental, immune, and behavioural disorders [7,8,9]. The human kinome is made of 518 protein kinases, and it is currently the largest druggable group in pharmacology [10]. The PKs family has become one of the most important drug targets over the past two decades. In fact, the U.S. Food and Drug Administration (FDA) has approved 66 small-molecule protein kinase inhibitors (PKIs) and more than 200 additional PKIs are under clinical trials [11].

Within trypanosomatids, the kinomes of *Leishmania major*, *L. infantum*, *L. braziliensis*, and *L. mexicana* are composed of 196, 224, 221, and 204 putative PKs, respectively, all of them belonging to the Ser/Thr PKs, except some dual-specificity PKs, with a complete absence of Tyr PKs, as described for all the trypanosomatids [12,13]. In most cases, the phosphorylation of a given protein is highly dependent on the stage of the parasite [14]. In *Leishmania*, PKs participate in the control of essential processes, such as the inter-stage differentiation, macrophage invasion, response to the stress, intracellular survival within the host, or drug resistance, among others [15,16,17].

The divergence among the human and *Leishmania* kinomes supports PKs as a relevant target for new anti-infective drugs with lower undesirable effects [18]. For this goal, a targeted PK must play an essential role in *Leishmania*, validated by genetic or pharmacological tools. A variety of different genetic techniques have been used for this purpose in *Leishmania* [19,20]. Using the CRISPR-Cas9 (clustered regularly interspaced short palindromic repeats-associated protein 9) technology, a comprehensive study of the whole *L. mexicana* kinome was recently reported [13]. In this relevant work, 43 PKs (21% of the kinome of this *Leishmania* species) were dubbed as essential for *Leishmania*, according to the impossibility of obtaining their viable knockout parasites. 

The pharmacological validation of specific inhibitors of a given PK must be assayed for leishmanicidal activity [21]. The quest for novel PKIs encompasses drug repurposing for PKIs against human PKs, as well as the screening of compound libraries [6,21]. Cross-inhibition with unexpected PKs in *Leishmania*, unforeseen off-targets, or effect on host PKs are some of the caveats inherent to the last approach. 

Among the validated essential PKs in *Leishmania* are Akt-like [22], CK1.2 (Casein Kinase 1.2) [19], Aurora kinase [23], or GSK-3 (Glycogen synthase kinase 3) [24,25]. GSK-3 is a multitask Ser/Thr PK, ubiquitous in eukaryotes, that works as a hub for multiple signal transduction pathways through the integration of a wide variety of intracellular stimuli, with ensuing effects on cell metabolism, division, and differentiation [26]. Its dysfunction underlies highly relevant human pathologies, such as cancer, diabetes type II, or neurodegenerative diseases [27,28,29,30]. 

The essential role of GSK-3 in trypanosomatids, was initially described for *Trypanosoma brucei* [24] and further extended to *Leishmania* after its genetic and pharmacological validation [13,25]. As in *T. brucei*, *Leishmania* GSK-3 is expressed as short and long forms, that differs in 150 amino acids at its *C*-terminus. Only the short form (GSK-3s) was subjected to pharmacological studies, due to its higher relevance over the long one in trypanosomatid survival [24]. Sequence identity between human GSK-3β (hGSK-3β) and *L. major* GSK-3 (LmjGSK-3s) was 41% [31]; only few residues lining the active site differed among these two enzymes. The induction of the apoptotic death of the parasite by 6-bromo-5-methylindirubin-3′-oxime, a specific inhibitor of LmjGSK-3s, was partially impeded by overexpression of the episomal gene supporting its validation as an essential target [25]. This was further confirmed after the failure to obtain viable knockout parasites for this enzyme [13]. The sequence of GSK-3 is highly preserved among *Leishmania* species; *L. major* shows 98% similarity of the amino acid sequence with *L. donovani*; and it is 100% identical to *L. infantum* [32]. Therefore, inhibitors of this enzyme may potentially work on the different forms of the disease, increasing the prospects for their pharmacological development.

A number of different inhibitory scaffolds were described as PKIs for this enzyme including indirubin analogues, the paullone alsteroaullone [33], and in a recent publication [34], benzoimidazole, oxadiazole, thiadiazolidinone, halomethylketone, and maleimide derivatives, the last two presumably acting as irreversible inhibitors, were added to this growing list. All of them act on the ATP-binding site of the *Leishmania donovani* GSK-3s (LdGSK-3s) and showed partial cross-inhibition with the human enzyme.

Altogether, we were prompted to search specific LdGSK-3 inhibitors as a new avenue for *Leishmania* chemotherapy, first by an unbiased study of the druggability of the binding sites of this enzyme that, in a second step, were further interrogated by our in-house chemical library using a two-pronged computational and experimental approach. From this, new quinones with selective inhibitory activity and active on *Leishmania* enzyme were found.

## 2. Materials and Methods

### 2.1. Chemical Procedures

Substrates were purchased from commercial sources. Melting points (Mp) were determined with a MP70 (Mettler Toledo España, Cornellá de Llobregat, Spain) apparatus. Flash column chromatography was carried out at medium pressure using silica gel (Grade 60, particle size 0.040–0.063 mm, 230–240 mesh ASTM; E. Merck, Darmstadt, Germany) with the indicated solvents as eluent (Hex = hexane; AcOEt = ethyl acetate; MeOH = methanol). Compounds were detected by UV absorption at 254 nm. The Bruker AVANCE-300 spectrometer (Bruker España, Madrid, Spain) was used to obtain both ^1^H NMR and ^13^C NMR spectra. For ^1^H NMR experiments, the spectrometer operated at 300 MHz, with typical spectral parameters: spectral width 15 ppm, pulse width 9 μs (57°), data size 32 K. For ^13^C NMR experiments, the same spectrometer operated at 75 MHz, and with the following acquisition parameters: spectral width 16 kHz, acquisition time 0.99 s, pulse width 9 μs (57°), data size 32 K. Chemical shifts (δ) are reported in values (ppm) relative to internal Me_4_Si and coupling constant *(J*) values in Hz (multiplicity of signals: s = singlet; d = doublet; t = triplet; q = quadruplet; m = multiplet; dd = doublet of doublets; td = triplet of doublets; sex = sextuplet). The high-resolution mass spectrometry (HRMS) analysis was obtained on an Agilent 1200 Series LC system (Agilent Technologies Spain, Las Rozas Spain), equipped with a binary pump, an autosampler, and a column oven, coupled to a 6520 quadrupole time-of-flight (Q-TOF) mass spectrometer. The mobile phase was acetonitrile:water (75:25, *v*:*v*) at a flow of 0.2 mL min^−1^. The ionization source was an electrospray ionization (ESI) interface working in the positive-ion mode. The electrospray voltage was set at 4.5 kV, the fragmentor voltage at 150 V and the drying gas temperature at 300 °C. Nitrogen (99.5% purity) was used both for nebulization (207 kPa) as well as drying gas (6 L min^−1^). Elemental analysis results were carried out on an Heraeus CHN-O-rapid analyser (Heraeus SA, Boadilla del Monte, Spain) at the analysis platform of Research Assistance Centre of the Complutense University of Madrid. For all the compounds, divergence from the theoretical values were within ±0.4% of the respective theoretical values. 

#### 2.1.1. Synthesis of Carbamate Derivatives **1–5**

The synthesis of carbamate derivatives **1–5** by the general procedure previously described [35] was followed.

KNCO (1.5 equiv.) was suspended in a mixture of anhydrous DMSO (4 mL) plus 1 mL of the corresponding alcohol, then 2,3-dichloro-1,4-naphthoquinone (1.0 equiv) was added. The mixture was stirred for 24 h at r. t. Excess of H_2_O (about 10 mL) was added and the mixture cooled (0–5 °C) for 30 min. The dark yellow-orange precipitate was filtered off and washed with H_2_O. The corresponding product was purified by flash column chromatography using as eluent solvent mixtures as indicated.

**Ethyl-(3-chloro-1,4-naphthoquinone-2-yl)carbamate (1)**. Reagents: KNCO (0.161 g, 1.98 mmol), 2,3-dichloro-1,4-naphthoquinone (0.300 g, 1.32 mmol), ethanol (1 mL, 17.04 mmol). Purification: Hex/AcOEt (90:10) Yield: 0.268 g, 73%. Yellow solid. Mp: 147–149 °C (lit. [35] 146–148 °C). ^1^H NMR (300 MHz, CDCl_3_) δ 8.19–7.99 (m, 2H), 7.79–7.63 (m, 2H), 7.14 (s, 1H, NH), 4.22 (q, *J* = 7.1 Hz, 2H), 1.28 (t, *J* = 7.1 Hz, 3H). ^13^C NMR (75 MHz, CDCl_3_) δ 178.3, 176.6, 150.0, 138.5, 133.7, 133.1, 130.4, 130.3, 129.3, 126.5, 126.1, 61.9, 13.3. Elemental analysis (C_13_H_10_ClNO_4_)—Calculated: C 55.83%, H 3.60%, N 5.01%. Found: C 56.04%, H 3.73%, N 5.13%. 

**Isopropyl-(3-chloro-1,4-naphthoquinone-2-yl)carbamate (2).** Reagents: KNCO (0.161 g, 1.98 mmol), 2,3-dichloro-1,4-naphthoquinone (0.300 g, 1.32 mmol), isopropanol (1 mL, 13.08 mmol). Purification: Hex/AcOEt (95:5). Yield: 0.081 g, 21%. Yellow solid. Mp: 149–151 °C (lit. [35] 148–149 °C). ^1^H NMR (300 MHz, CDCl_3_) δ 8.15–7.96 (m, 2H), 7.77–7.61 (m, 2H), 7.15 (s, 1H, NH), 4.95 (hept, *J* = 6.3 Hz, 1H), 1.25 (d, *J* = 6.3 Hz, 6H). ^13^C NMR (75 MHz, CDCl_3_) δ 178.3, 176.6, 149.6, 138.7, 133.7, 133.0, 130.4, 130.1, 129.3, 126.4, 126.1, 69.9, 20.9 (2C). Elemental analysis (C_14_H_12_ClNO_4_)—Calculated: C 57.25%, H 4.12%, N 4.77%. Found: C 56.97%, H 4.13%, N 4.96%. 

**Benzyl-(3-chloro-1,4-naphthoquinone-2-yl)carbamate (3).** Reagents: KNCO (0.120 g, 1.5 mmol), 2,3-dichloro-1,4-naphthoquinone (0.227 g, 1 mmol), Benzyl alcohol (1 mL, 9.7 mmol). Purification: Hex/AcOEt (80:20). Yield: 0.24 g, 70%. Yellow solid. Mp: 163–164 °C. ^1^H NMR (300 MHz, CDCl_3_) δ 8.20–8.14 (m, 1H), 8.12–8.06 (m, 1H), 7.80–7.72 (m, 2H), 7.44–7.35 (m, 5H), 7.33 (s, 1H, NH), 5.24 (s, 2H). ^13^C NMR (75 MHz, CDCl_3_) δ 179.3, 177.7, 151.0, 139.5, 135.1, 134.9, 134.3, 131.7, 131.5, 130.4, 128.8 (3C), 128.7 (2C), 127.6, 127.2, 68.7. HRMS (ESI/Q-TOF) *m*/*z*: [M + H]^+^—Calculated for C_18_H_13_ClNO_4_ 342.0533. Found: 342.0539. 

**4-Methoxybenzyl-(3-chloro-1,4-naphthoquinone-2-yl)carbamate (4).** Reagents: KNCO (0.120 g, 1.5 mmol), 2,3-dichloro-1,4-naphthoquinone (0.227 g, 1 mmol), 4-methoxybenzyl alcohol (1 mL, 8 mmol). Purification: CH_2_Cl_2_/MeOH (99:1). Yield: 0.12 g, 34%. Yellow solid. Mp: 137–147 °C. ^1^H NMR (300 MHz, CDCl_3_) δ 8.21–8.14 (m, 1H), 8.13–8.07 (m, 1H), 7.81–7.71 (m, 2H), 7.36 (d, *J* = 8.7 Hz, 2H), 7.27 (s, 1H, NH), 6.91 (d, *J* = 8.7 Hz, 2H), 5.18 (s, 2H), 3.81 (s, 3H). ^13^C NMR (75 MHz, CDCl_3_) δ 179.4, 177.8, 160.1, 151.1, 139.6, 134.9, 134.3, 131.9, 131.6, 130.7 (2C), 130.4, 127.6, 127.3, 124.0, 114.2 (2C), 68.6, 55.5. HRMS (ESI/Q-TOF) *m*/*z*: [M + Na]^+^—Calculated for C_19_H_14_ClNO_5_Na 394.0458. Found: 394.0488. 

**3,4,5-Trimethoxybenzyl-(3-chloro-1,4-naphthoquinone-2-yl)carbamate (5).** Reagents: KNCO (0.12 g, 1.5 mmol), 2,3-dichloro-1,4-naphthoquinone (0.227 g, 1 mmol), 3,4,5-trimethoxybenzyl alcohol (1 mL, 6.2 mmol). Purification: Hex/AcOEt (80:20). Yield: 0.09 g, 21%. Yellow solid. Mp: 167–168 °C. ^1^H NMR (300 MHz, CDCl_3_) δ 8.20–8.13 (m, 1H), 8.12–8.06 (m, 1H), 7.81–7.72 (m, 2H), 7.34 (s, 1H, NH), 6.63 (s, 2H), 5.16 (s, 2H), 3.87 (s, 6H), 3.84 (s, 3H). ^13^C NMR (75 MHz, CDCl_3_) δ 179.3, 177.7, 153.5 (2C), 151.0, 139.5, 138.3, 134.9, 134.3, 131.8, 131.5, 130.7, 130.3, 127.6, 127.2, 105.8 (2C), 69.0, 61.0, 56.3 (2C). HRMS (ESI/Q-TOF) *m*/*z*: [M + Na]^+^—Calculated for C_21_H_18_ClNO_7_Na 454.0669. Found: 454.0639.

#### 2.1.2. General Procedure for the Synthesis of Amines **6–13**

2,3-Dichloro-1,4-naphthoquinone (1 equiv.) was dissolved in 4 mL of DMSO, and the corresponding amine (1.5 equiv.) was added dropwise at r. t. The mixture was stirred for 10 min at r. t., excess of H_2_O (about 10 mL) was added, and the mixture cooled (0–5 °C) for 30 min. The precipitate, with a bright-red colour in most of the cases, was filtered off and washed with H_2_O. The residue was purified by flash column chromatography using as eluent solvent mixtures as indicated. 

**2-Chloro-3-(propylamino)-1,4-naphthoquinone (6).** Reagents: *N*-propylamine (0.12 mL, 1.5 mmol), 2,3-dichloro-1,4-naphthoquinone (0.227 g, 1 mmol). Purification: Hex/AcOEt (80:20). Yield: 0.23 g, 96%. Bright-red solid. Mp: 118–119 °C. ^1^H NMR (300 MHz, CDCl_3_) δ 8.15 (dd, *J* = 7.7, 0.9 Hz, 1H), 8.03 (dd, *J* = 7.6, 0.9 Hz, 1H), 7.72 (td, *J* = 7.5, 1.4 Hz, 1H), 7.62 (td, *J* = 7.5, 1.4 Hz, 1H), 6.09 (s, 1H, NH), 3.82 (q, *J* = 6.3 Hz, 2H), 1.72 (sex, *J* = 7.3 Hz, 2H), 1.01 (t, *J* = 7.4 Hz, 3H). ^13^C NMR (75 MHz, CDCl_3_) δ 180.7, 177.0, 144.4, 135.1, 133.0, 132.5, 129.9, 126.9 (2C), 110.4, 46.8, 24.4, 11.3. HRMS (ESI/Q-TOF) *m*/*z*: [M + H]^+^—Calculated for C_13_H_13_ClNO_2_ 250.0635. Found: 250.0633. 

**2-Chloro-3-(pentylamino)-1,4-naphthoquinone (7).** Reagents: *N*-pentylamine (0.17 mL, 1.5 mmol), 2,3-dichloro-1,4-naphthoquinone (0.227 g, 1 mmol). Purification: Hex/AcOEt (70:30). Yield: 0.25 g, 82%. Bright-red solid. Mp: 99–100 °C. ^1^H NMR (300 MHz, CDCl_3_) δ 8.15 (dd, *J* = 7.6, 1.0 Hz, 1H), 8.03 (dd, *J* = 7.6, 1.0 Hz, 1H), 7.72 (td, *J* = 7.6 Hz, 1.4 Hz, 1H), 7.62 (td, *J* = 7.6, 1.4 Hz, 1H), 6.08 (s, 1H, NH), 3.85 (q, *J* = 6.3 Hz, 2H), 1.69 (m, 2H), 1.30–1.40 (m, 4H), 0.93 (t, *J* = 7.3, 3H). ^13^C NMR (75 MHz, CDCl_3_) δ 180.7, 176.9, 144.3, 135.1, 132.9, 132.5, 129.8, 127.0, 126.9, 110.3, 45.1, 30.8, 28.9, 22.5, 14.1. HRMS (ESI/Q-TOF) *m*/*z*: [M + H]^+^—Calculated for C_15_H_17_ClNO_2_ 278.0948. Found: 278.0951.

**2-(*tert*-Butylamino)-3-chloro-1,4-naphthoquinone (8).** Reagents: *tert*-butylamine (0.16 mL, 1.5 mmol), 2,3-dichloro-1,4-naphthoquinone (0.227 g, 1 mmol). Purification: Hex/AcOEt (70:30). Yield: 0.21 g, 81%. Dark-red solid. Mp: 101–102 °C. ^1^H NMR (300 MHz, CDCl_3_) δ 8.12 (dd, *J* = 7.4, 1.4 Hz, 1H), 8.03 (dd, *J* = 7.6, 1.9 Hz, 1H), 7.71 (td, *J* = 7.5, 1.4 Hz, 1H), 7.62 (td, *J* = 7.4, 1.3 Hz, 1H), 6.10 (s, 1H, NH), 1.57 (s, 9H). ^13^C NMR (75 MHz, CDCl_3_) δ 181.0, 177.9, 145.9, 134.8, 132.6, 132.4, 130.4, 127.0, 126.8, 112.5, 55.2, 31.8 (3C). HRMS (ESI/Q-TOF) *m*/*z*: [M + H]^+^—Calculated for C_14_H_15_ClNO_2_ 264.0791. Found: 264.0793. 

**2-Chloro-3-(cyclohexylamino)-1,4-naphthoquinone (9).** Reagents: cyclohexylamine (0.17 mL, 1.5 mmol), 2,3-dichloro-1,4-naphthoquinone (0.227 g, 1 mmol). Purification: Hex/AcOEt (90:10). Yield: 0.27 g, 93%. Bright-red solid. Mp: 122–123 °C. ^1^H NMR (300 MHz, CDCl_3_) δ 8.14 (dd, *J* = 7.6, 1.0 Hz, 1H), 8.02 (dd, *J* = 7.6, 1.0 Hz, 1H), 7.71 (td, *J* = 7.6, 1.4 Hz, 1H), 7.61 (td, *J* = 7.5, 1.4 Hz, 1H), 6.04 (s, 1H, NH), 4.50–4.34 (m, 1H), 2.16–2.00 (m, 2H), 1.86–1.73 (m, 2H), 1.73–1.62 (m, 1H), 1.49–1.18 (m, 5H). ^13^C NMR (75 MHz, CDCl_3_) δ 180.8, 176.9, 143.5, 135.0, 133.0, 132.5, 130.0, 127.0 (2C), 110.1, 52.6, 34.8 (2C), 25.5, 24.6 (2C). HRMS (ESI/Q-TOF) *m*/*z*: [M + H]^+^—Calculated for C_16_H_17_ClNO_2_ 290.0948. Found: 290.0939. 

**2-Chloro-3-((3,4,5-trimethoxybenzyl)amino)-1,4-naphthoquinone (10).** Reagents: 3,4,5-trimethoxybenzyl amine (0.26 mL, 1.5 mmol), 2,3-dichloro-1,4-naphthoquinone (0.227 g, 1 mmol). Purification: Hex/AcOEt (80:20). Yield: 0.35 g, 93%, Bright-red solid. Mp: 161–163 °C. ^1^H NMR (300 MHz, CDCl_3_) δ 8.16 (dd, *J* = 7.6, 1.0 Hz, 1H), 8.04 (dd, *J* = 7.6, 1.0 Hz, 1H), 7.74 (td, *J* = 7.6, 1.4 Hz, 1H), 7.64 (td, *J* = 7.6, 1.4 Hz, 1H), 6.56 (s, 2H), 6.16 (s, 1H, NH), 4.98 (d, *J* = 5.8 Hz, 2H), 3.86 (s, 6H), 3.85 (s, 3H). ^13^C NMR (75 MHz, CDCl_3_) δ 180.6, 177.1, 153.9 (2C), 144.2, 138.1, 135.2, 133.5, 132.8, 132.7, 130.0, 127.1, 127.0, 111.2, 105.1 (2C), 61.0, 56.4 (2C), 49.6. HRMS (ESI/Q-TOF) *m*/*z*: [M + H]^+^—Calculated for C_20_H_19_ClNO_5_ 388.0952. Found: 388.0943. 

**2-Chloro-3-((4-chlorobenzyl)amino)-1,4-naphthoquinone (11).** Reagents: 4-chloro-benzylamine (0.13 mL, 1.5 mmol), 2,3-dichloro-1,4-naphthoquinone (0.227 g, 1 mmol). Purification: Hex/AcOEt (80:20). Yield: 0.28 g, 85%. Bright-red solid. Mp: 162–163 °C. ^1^H NMR (300 MHz, CDCl_3_) δ 8.15 (dd, *J* = 7.7, 1.0 Hz, 1H, H8), 8.04 (dd, *J* = 7.7, 1.0 Hz, 1H), 7.74 (td, *J* = 7.6, 1.4 Hz, 1H), 7.64 (td, *J* = 7.6, 1.4 Hz, 1H), 7.35 (d, *J* = 8.5 Hz, 2H), 7.27 (d, *J* = 8.5 Hz, 2H), 6.20 (s, 1H, NH), 5.02 (d, *J* = 5.0 Hz, 2H). ^13^C NMR (75 MHz, CDCl_3_) δ 180.5, 177.1, 144.1, 136.7, 135.2, 132.8, 132.7, 130.0, 129.4 (2C), 129.1 (2C), 128.0, 127.1 (2C), 111.9, 48.3. HRMS (ESI/Q-TOF) *m*/*z*: [M + H]^+^—Calculated for C_17_H_12_Cl_2_NO_2_ 332.0245. Found: 332.0243. 

**2-Chloro-3-((4-chlorophenyl)amino)-1,4-naphthoquinone (12).** Reagents: 4-chloro aniline (0.19 g, 1.5 mmol), 2,3-dichloro-1,4-naphthoquinone (0.227 g, 1 mmol). Purification: Hex/AcOEt (85:15). Yield: 0.29 g, 92%. Dark-red solid. Mp: 265–266 °C. ^1^H NMR (300 MHz, CDCl_3_) δ 8.20 (dd, *J* = 7.5, 1.0 Hz, 1H), 8.13 (dd, *J* = 7.5, 1.1 Hz, 1H), 7.79 (td, *J* = 7.5, 1.4 Hz, 1H), 7.71 (td, *J* = 7.5, 1.4 Hz, 1H), 7.61 (s, 1H, NH), 7.32 (d, *J* = 8.7 Hz, 2H), 7.01 (d, *J* = 8.4 Hz, 2H). ^13^C NMR (75 MHz, CDCl_3_) δ 180.6, 176.8, 142.0, 141.4, 136.2, 135.3, 133.3, 132.6, 131.2, 128.7 (2C), 127.4, 127.2, 125.5 (2C), 110.7. HRMS (ESI/Q-TOF) *m*/*z*: [M + H]^+^—Calculated for C_16_H_10_Cl_2_NO_2_ 318.0089. Found: 318.0088. 

**2-(Benzylamino)-3-chloro-1,4-naphthoquinone (13).** Reagents: benzylamine (0.16 mL, 1.5 mmol), 2,3-dichloro-1,4-naphthoquinone (0.227 g, 1 mmol). Purification: Hex/AcOEt (85:15). Yield: 0.26 g, 87%. Bright-red solid. Mp: 109–110 °C. ^1^H NMR (300 MHz, CDCl_3_) δ 8.15 (dd, *J* = 7.6, 1.0 Hz, 1H), 8.03 (dd, *J* = 7.6, 1.0 Hz, 1H), 7.72 (td, *J* = 7.6, 1.4 Hz, 1H), 7.62 (td, *J* = 7.6, 1.4 Hz, 1H), 7.42–7.29 (m, 5H), 6.23 (s, 1H, NH), 5.05 (d, *J* = 6.0 Hz, 2H). ^13^C NMR (75 MHz, CDCl_3_) δ 180.6, 177.0, 144.2, 138.0, 135.1, 132.8, 132.7, 130.0, 129.2 (3C), 128.2 (2C), 127.8, 127.0, 111.5, 49.1. HRMS (ESI/Q-TOF) *m*/*z*: [M + H]^+^—Calculated for C_17_H_13_ClNO_2_ 298.0635. Found: 298.0624.

#### 2.1.3. Procedure for the Synthesis of 1-(*tert*-Butyl)-3-(3-chloro-1,4-naphthoquinone-2-yl)urea (**14**)

KNCO (0.12 g, 1.5 mmol) was resuspended in a mixture of anhydrous DMSO (4 mL), and *tert*-butylamine (0.16 mL, 1.5 mmol). Then, 2,3-dichoro-1,4-naphthoquinone (0.227 g, 1 mmol) was added. The mixture was stirred for 24 h at r. t., excess of H_2_O (about 10 mL) was added, and the mixture cooled (0–5 °C) for 1 h. The dark precipitate was filtered off and washed with cold H_2_O. The aqueous phase was extracted with EtOAc (3 × 10 mL), and the solvent evaporated. The product was purified using a silica gel column chromatography with a mixture Hex/AcOEt 80:20 as eluent to afford 0.16 g (51.6% yield) of the urea derivative compound as a yellow solid. Mp: 195–196 °C. ^1^H NMR (300 MHz, CDCl_3_) δ 8.09 (dd, *J* = 7.4, 1.4 Hz, 1H), 8.01 (dd, *J* = 7.6, 1.9 Hz, 1H), 7.72–7.60 (m, 2H), 7.09 (s, 1H), 5.09 (s, 1H), 1.35 (s, 9H). ^13^C NMR (75 MHz, CDCl_3_) 180.2, 177.8, 150.3, 141.1, 134.7, 133.8, 131.9, 130.4, 127.4, 127.4, 127.1, 51.7, 29.0 (3C). HRMS (ESI/Q-TOF) *m*/*z*: [M + H]^+^—Calculated for C_15_H_16_ClN_2_O_3_ 307.0849. Found: 307.0829. 

#### 2.1.4. Procedure for the Synthesis of 2-Amino-1,4-naphthoquinone (**15**)

To a solution of 2-bromo-1,4-naphthoquinone (1 g, 4.2 mmol) in THF, 32% aqueous NH_3_ (2.5 mL, 42 mmol) was added, then the mixture was stirred for 24 h at r. t. The solvent was evaporated under vacuum. The residue was purified by flash column chromatography with a mixture Hex/AcOEt 80:20 as eluent to afford 0.33 g (45% yield) as an orange solid. Mp: 206–207 °C (lit. [36] 202–204 °C). HRMS (ESI/Q-TOF) *m*/*z*: [M + H]^+^—Calculated for C_10_H_8_NO_2_: 174.0555. Found: 174.0546. 

#### 2.1.5. General Procedure for the Synthesis of Carbamates **16–17**

2-Amino-1,4-naphthoquinone (1 equiv.) and NaH (3 equiv. 60% dispersion in mineral oil) were suspended in anhydrous DMF. Then, the corresponding chloroformate (1.5 equiv.) was added dropwise continuously under inert Ar atmosphere. The reaction mixture was stirred for 15 min at r. t., excess of H_2_O (about 10 mL) was added, and the mixture is extracted with CH_2_Cl_2_ (3 × 10 mL). The organic phases are collected and then dried over anhydrous MgSO_4_. The desiccant was filtered, and the organic solvent removed under vacuum. The precipitate was purified by flash column chromatography using as eluent solvent mixtures as indicated to obtain the desired products. 

**Methyl-(1,4-naphthoquinone-2-yl)carbamate (16).** Reagents: 2-amino-1,4-naphthoquinone (0.1 g, 0.6 mmol), NaH (0.14 g, 3.6 mmol, 60% dispersion in mineral oil), methyl chloroformate (71 µL, 0.9 mmol). Purification: Hex/AcOEt (70:30). Yield: 0.08 g, 57%. Light yellow solid. Mp: 201–202 °C. ^1^H NMR (300 MHz, CDCl_3_) δ 8.10 (dd, *J* = 7.8, 1.3 Hz, 2H), 7.89 (s, 1H, NH), 7.78 (td, *J* = 7.5, 1.5 Hz, 1H), 7.71 (td, *J* = 7.4, 1.5 Hz, 1H), 7.51 (s, 1H), 3.85 (s, 3H). ^13^C NMR (75 MHz, CDCl_3_) δ 185.9, 180.7, 152.9, 140.9, 135.1, 133.3, 132.4, 130.2, 126.8, 126.6, 115.6, 53.4. HRMS (ESI/Q-TOF) *m*/*z*: [M + H]^+^—Calculated for C_12_H_10_NO_4_ 232.0610. Found: 232.0613. 

**Ethyl-(1,4-naphthoquinone-2-yl)carbamate (17).** Reagents: 2-amino-1,4-naphthoquinone (0.1 g, 0.6 mmol), NaH (0.14 g, 3.6 mmol, 60% dispersion in mineral oil), ethyl chloroformate (85 µL, 0.9 mmol). Purification: Hex/AcOEt (70:30). Yield: 0.07 g, 47%. Light yellow solid. Mp: 162–163 °C. ^1^H NMR (300 MHz, CDCl_3_) δ 8.10 (dd, *J* = 7.8, 1.3 Hz, 2H, H5, H8), 7.85 (s, 1H, NH), 7.78 (td, *J* = 7.5, 1.5 Hz, 1H), 7.71 (td, *J* = 7.4, 1.5 Hz, 1H), 7.51 (s, 1H), 4.29 (q, *J* = 7.1 Hz, 2H), 1.35 (t, *J* = 7.1 Hz, 3H). ^13^C NMR (75 MHz, CDCl_3_) δ 184.9, 180.7, 152.5, 141.0, 135.1, 133.3, 132.4, 130.3, 126.6, 126.6, 115.5, 62.6, 14.5. HRMS (ESI/Q-TOF) *m*/*z*: [M + H]^+^—Calculated for C_13_H_12_NO_4_ 246.0766. Found: 246.0749.

#### 2.1.6. General Procedure for the Synthesis of Amides **18–20**

2-Amino-1,4-naphthoquinone (1 equiv.) and NaH (3 equiv. 60% dispersion in mineral oil) were dissolved in anhydrous DMF. Then, an excess of the corresponding acyl chloride was added dropwise continuously (1.5 equiv.). The reaction mixture was stirred for 30 min at r. t. Afterwards, excess of H_2_O (about 10 mL) was added, and the mixture was extracted with CH_2_Cl_2_ (3 × 10 mL) and washed with 1M NaOH and 1M HCl. The organic phases were collected and then dried over anhydrous MgSO_4_. The desiccant was filtered, and the organic solvent removed under vacuum. The reaction crude was purified by flash column chromatography using as eluent solvent mixtures as indicated to obtain the desired products. 

***N-(*1,4-Naphthoquinone-2-yl)acetamide (18).** Reagents: acetyl chloride (32 µL, 0.45 mmol), 2-amino-1,4-naphthoquinone (0.05 g, 0.3 mmol), NaH (0.04 g, 0.9 mmol, 60% dispersion in mineral oil). Purification: CH_2_Cl_2_/MeOH (90:10). Yield: 0.046 g, 71%. Yellow solid. Mp: 201–203 °C. ^1^H NMR (300 MHz, CDCl_3_) δ 8.36 (s, 1H, NH), 8.13–8.11 (m, 1H), 8.10–8.08 (m, 1H), 7.85 (s, 1H), 7.79 (td, *J* = 7.5, 1.5 Hz, 1H), 7.72 (td, *J* = 7.5, 1.5 Hz, 1H), 2.29 (s, 3H). ^13^C NMR (75 MHz, CDCl_3_) δ 185.4, 181.2, 169.5, 140.0, 135.2, 133.4, 132.3, 130.1, 126.8, 126.6, 117.4, 25.2. HRMS (ESI/Q-TOF) *m*/*z*: [M + H]^+^—Calculated for C_12_H_10_NO_3_ 216.0661. Found: 216.0663. 

***N*-(1,4-Naphthoquinone-2-yl)propionamide (19).** Reagents: propionyl chloride (40 µL, 0.45 mmol), 2-amino-1,4-naphthoquinone (0.05 g, 0.3 mmol), NaH (0.04 g, 0.9 mmol, 60% dispersion in mineral oil). Purification: CH_2_Cl_2_/MeOH (90:10). Yield: 0.016 g, 24%. Yellow solid. Mp: 173–175 °C. ^1^H NMR (300 MHz, CDCl_3_) δ 8.35 (s, 1H, NH), 8.09 (dd, *J* = 7.4, 1.7 Hz, 2H), 7.85 (s, 1H), 7.78 (td, *J* = 7.5, 1.5 Hz, 1H), 7.71 (td, *J* = 7.5, 1.5 Hz, 1H), 2.52 (q, *J* = 7.5 Hz, 2H), 1.02 (t, *J* = 7.5 Hz, 3H). ^13^C NMR (75 MHz, CDCl_3_) δ 185.4, 181.3, 173.2, 140.1, 135.1, 133.4, 132.4, 130.1, 126.8, 126.5, 117.2, 31.3, 9.2. HRMS (ESI/Q-TOF) *m*/*z*: [M + H]^+^—Calculated for C_13_H_12_NO_3_ 230.0817. Found: 230.0823. 

***N*-(1,4-Naphthoquinone-2-yl) phenylacetamide (20).** Reagents: phenylacetyl chloride (119 µL, 0.9 mmol), 2-amino-1,4-naphthoquinone (0.1 g, 0.6 mmol), NaH (0.08 g, 1.8 mmol, 60% dispersion in mineral oil). Purification: CH_2_Cl_2_/MeOH (90:10). Yield: 0.07 g, 27%. Dark yellow solid. Mp: 130–132 °C. ^1^H NMR (300 MHz, CDCl_3_) δ 8.43 (s, 1H, NH), 8.09 (dd, *J* = 7.4, 1.7 Hz, 2H), 7.88 (s, 1H), 7.78 (td, *J* = 7.5, 1.3 Hz, 1H), 7.71 (td, *J* = 7.5, 1.3 Hz, 1H), 7.52–7.34 (m, 5H), 3.85 (s, 2H). ^13^C NMR (75 MHz, CDCl_3_) δ 185.2, 181.6, 171.8, 142.1, 138.2, 135.0, 133.2, 132.2, 130.3, 129.3 (3C), 128.2 (2C), 126.6, 126.5, 117.2, 45.0. HRMS (ESI/Q-TOF) *m*/*z*: [M + H]^+^—Calculated for C_18_H_14_NO_3_ 292.0974. Found: 292.0963.

### 2.2. Computational Studies

The only crystal structure available for LmjGSK-3s was retrieved [31] (Protein Data Bank (PDB) code: 3E3P). Nevertheless, the crystallized protein did not display the decapeptide loop located at the upper part of the ATP binding pocket. Therefore, this loop was modelled prior to the analysis using the Modeller 9.20 software (University of San Francisco, San Francisco, CA, USA) [37].

#### 2.2.1. Ligand Preparation

The preparation of the MBC chemical library and its 2D-to-3D conversion was carried out using the LigPrep [38] tool (*Schrödinger Release 2015-4*. Schrödinger, LLC: New York, NY, USA), with addition of hydrogen atoms and calculation of the ionization state of the molecules at physiological pH. All the molecules were desalted and minimized as default at the last step. The generation of possible tautomers and low-energy ring conformations, as well as a final energy minimization step were carried out using the OPLS-2005 force field [39,40].

#### 2.2.2. Protein Preparation

The Protein Preparation Wizard tool [41] implemented on Maestro (*Schrödinger Release 2015-4*. Schrödinger, LLC: New York, NY, USA) [42] was used to pre-process and refine the structures of the proteins by H-bond assignment and calculation of the protonation state of the residues at physiological pH with a final restraint minimization.

#### 2.2.3. Cavity Detection Analysis

The potential cavities on the different enzymes were identified using the Fpocket 2.0 software (Barcelona, Spain) [43] a pocket detection package, based on Voronoi tessellation and alpha spheres building. For that, the qhull package was used for Voronoi tessellation. After the structural analysis, the pockets of the protein were compiled and ranked according to the scores provided by the program. Further analyses were carried out based on the visual inspection of best ranked pockets. MDpocket, tool included in Fpocket 2.0, [44] was used to detect cavities for a set of PDB structures. A total number of 56 crystal structures of GSK-3 available at the moment of the study, mostly from human origin, were retrieved for the analysis (PDB codes: 1R0E, 1Q5K, 1J1B, 1J1C, 1PYX, 1Q3D, 1Q3W, 1Q41, 1Q4L, 1O9U, 1H8F, 1I09, 1GNG, 2O5K, 2OW3, 2JLD, 3L1S, 3E3P, 3DU8, 3F88, 3F7Z, 3I4B, 3GB2, 3PUP, 3M1S, 3Q3B, 3ZRM, 3ZRL, 3ZRK, 3SD0, 3SAY, 3ZDI, 4DIT, 4ACH, 4ACG, 4ACD, 4ACC, 4B7T, 4J71, 4J1R, 4IQ6, 4NU1, 4NM7, 4NM5, 4NM3, 4NM0, 4PTG, 4PTE, 4PTC, 4AFJ, 4E7W, 5F95, 5F94, 5HLN, 5K5N, 5AIR).

#### 2.2.4. Hotspots Maps

The Fragment Hotspot maps 0.11.0 software (CDCC Cambridge, UK) [45] was utilised to define the location and environment of the binding sites on the protein. After an initial calculation of atomic hotspots, the Fragment Hotspot maps were further produced using simple molecular probes. These maps uncover specifically fragment-binding sites and their respective pharmacophores. The interactions identified with the highest relevance were further employed to set up filters for virtual screening studies and the quest for molecules that fulfil these essential interactions. 

#### 2.2.5. Virtual Screening

Virtual screening was performed employing the Glide module [46] within the Schrödinger software package (*Schrödinger Release 2017-1*. Schrödinger, LLC: New York, NY, USA, with the corresponding 3D target structure and the MBC library [47]. In all cases, the centroid of the grid was taken as the centre of the catalytic or the specified pocket. For the grid generation a scaling factor of 1.0 in van der Waals radius scaling, and a partial charge cutoff of 0.25 were used. The virtual screening used either a standard precision (SP) or an extra precision (XP) mode [48]. The ligand sampling was flexible, epik state penalties were added, and a 2.5 kcal/mol energy window was used for ring sampling. The distance-dependent dielectric constant was 4.0 with a maximum number of minimization steps of 100,000 in the energy minimization step. In the clustering, poses were considered as duplicates and discarded if RMSD values were lower than 0.5 Å and the maximum atomic displacement lower than 1.3 Å. 

### 2.3. Experimental Biology Procedures

#### 2.3.1. Reagents

Commercial reagents were of the highest quality available and purchased from SIGMA-Aldrich Spain unless otherwise stated. Growth media (RPMI 1640 medium, RPMI 1640 without red phenol, and M199 medium) were obtained from Gibco (Life Technologies Europe, Bleiswij, The Netherlands).

#### 2.3.2. Assessment of Inhibition of GSK-3 with Kinase Glo^®^

Extraction, purification, and evaluation of compounds against recombinant LdGSK-3s was carried out as described. For that, a recombinant LdGSK-3s with a His-tag was obtained as inclusion bodies, that were further solubilized, and purified by chromatography first on a Ni^2+^ column an in a second step on a DEAE (diethylaminoethyl) ionic exchange column [34]. Samples were made in duplicate, and assays were repeated at least three times. IC_50_ (inhibitory concentration that decreases enzymatic activity by 50%) was calculated using the statistical module of SigmaPlot v11.0 software. Evaluation of compounds against hGSK-3β was performed using human recombinant GSK-3β purchased from Millipore as described previously [49]. Compounds were prepared as a 10 mM stock solution in DMSO. 

The mechanism of inhibition of LdGSK-3s by **1** and **2** was studied as described for hGSK-3β [49]. Briefly, kinetic experiments were carried out at four different concentrations of ATP (10, 5, 2.5, and 1 µM), in the absence or presence of the inhibitors, at either 2.5 or 5 µM, while the phospho-glycogen synthase peptide-2 (GS2) substrate was kept constant at 25 µM. The data were processed using Microsoft Excel software and results presented as Eadie–Scatchard plots (*v* vs. *v*/[ATP]).

#### 2.3.3. Cell

The axenic parasite strains used were *Leishmania donovani* promastigotes (MHOM/SD/00/1S-2D) and *L. pifanoi* (MHOM/VE/60/Ltrod) axenic amastigotes, as well as its derived-strain transfected with a pLEXSY expression vector containing the mCherry gene (mCherry-*L. pifanoi*). The growth medium for promastigotes was RPMI 1640 medium supplemented with 5 mM HEPES, 1.7 mM HCO_3_Na, 10% HIFCS (heat-inactivated fetal calf serum; Biowest, Nuaillé—France), 2 mM L-glutamine, 20 U/mL unicillin (ERN Laboratories, S.A., Barcelona, Spain), 24 µg/mL gentamicin (NORMON Laboratories, S.A., Tres Cantos, Spain); pH 6.8–6.9 (RPMI 1640-HIFCS). Axenic amastigotes were grown in M199-HIFCS (M199 medium supplemented with 20% HIFCS, 0.5% trypticase peptone (BD Biosciences, San Agustín de Guadalix, Spain), 14 mM D-glucose, 76.7 µM hemin, 5.1 mM glutamine, 40 µg/mL gentamicin; pH 7–7.2). Promastigotes and axenic amastigores were grown at 26 °C or 32 °C, respectively. 

#### 2.3.4. Cell Harvesting

*Leishmania* parasites were collected at late exponential growth phase by centrifugation at 1610× *g* at 4 °C. Elicited mouse peritoneal macrophages (MPM) were obtained from 8-week-old Balb/c mice through i.p. injection with 1 mL of 10% thioglycolate medium 72 h before extraction. Macrophages were obtained by peritoneal washing (10 mL PBS, 4 °C). After extraction, macrophages were cultured in RPMI 1640-HIFCS at 37 °C and 5% CO_2_. 

#### 2.3.5. Leishmanicidal and Cytotoxicity Assays of the Different Compounds

Both activities were assessed by inhibition of MTT (3-(4,5-dimethylthiazol-2-yl)-2,5-diphenyltetrazolium bromide) reduction, as described in [34]. Samples were made in triplicate and assays were repeated at least twice. DMSO content in the medium was adjusted to 0.25% *v*/*v* (final concentration), regardless of the final concentration of the drug, and for the control, the untreated parasites. IC*x* (inhibitory concentration that inhibits parasite growth or enzymatic activity by *x*%) was calculated using the statistical module of SigmaPlot v11.0 software. 

#### 2.3.6. Cytotoxicity against Intracellular Amastigotes

Mouse peritoneal macrophages resuspended in RPMI 1640-HIFCS were seeded in a 24-well plate (2 × 10^5^ cells/well) with sterile 14 mm-diameter coverslides placed at the bottom of each well. Cells were allowed to adhere to the glass surface of the coverslides (24 h, 37 °C, 5% CO_2_). Initial infection was carried out with mCherry–*L. pifanoi* (amastigote: macrophage ratio 3:1) at 32 °C for 4 h in RPMI 1640-HIFCS. After removal of the non-phagocytized amastigotes (3 times wash with 1 mL warm phosphate-buffered saline PBS) the infection was allowed to progress at 32 °C for another 24 h. Next, cells were incubated with each compound at 32 °C for 20 h. The infection index (number of amastigotes per macrophage) was evaluated by fluorescence microscopy (Leica DIALUX 20, Leica Microsystem, L’Hospitalet de Llobregat, Spain). At least 6 different fields gathering up to 100 macrophages were counted in each preparation. Statistical significance was calculated by *p* values using Student’s *t*-test. Each sample was made by duplicate and experiments were repeated at least twice.

#### 2.3.7. Mitochondrial Membrane Depolarization

Variation of the mitochondrial membrane polarization was evaluated by the intracellular accumulation of rhodamine 123 (Rh123) [50]. *Leishmania* parasites at 20 × 10^6^ cells/mL in Hanks buffered salt solution supplemented with 10 mM D-glucose (HBSS-Glc) were incubated with the compounds for 4 h either at 26 °C or 32 °C for promastigotes or amastigotes, respectively. Then, cells were washed by centrifugation (4 min, 13,000× *g*, 4 °C) and the cells resuspended in 100 μL HBSS-Glc plus 0.3 μg/mL Rh123, incubated for 10 min in darkness. Extracellular Rh123 was removed by washing in cold HBSS-Glc. Lastly, the cells were resuspended in 900 μL HBSS-Glc and measured in a Coulter XL EPICS flow cytometer (λ_EXC_ = 488 nm, λ_EM_ = 520 nm). 

Samples were prepared by duplicate and assays were repeated at least twice. The control for depolarization consisted of parasites incubated with 20 mM KCN for 40 min prior to Rh123 incubation.

#### 2.3.8. Oxygen Consumption Rate

Variation in the O_2_ consumption rate of *L. donovani* promastigotes was measured in a Clark electrode (Hansatech Instruments, King’s Lynn, UK) at 1 × 10^8^ cells/mL in 600 μL of respiration buffer (10 mM Tris-HCl, 125 mM sucrose, 65 mM KCl, 1 mM MgCl_2_, 2.5 mM NaH_2_PO_4_, 0.3 mM EGTA, 5 mM succinic acid; pH 7.2). Compounds were added at 100 × final concentration to whole parasites, and oxygen consumption was monitored for 8–10 min. 

#### 2.3.9. Measurement of Sub G_0_/G_1_ Population

*L. donovani* promastigotes (2 × 10^6^ cells/mL) in RPMI-HIFCS were incubated with each compound in 24-well plates for 72 h at 26 °C by duplicate. Next, compounds were removed by washing with HBSS-Glc (15,700× *g*, 4 min, 4 °C) and cells were resuspended in 15 mL of HBSS-Glc, fixed and permeabilized by addition of 200 μL 70% cold EtOH and incubated overnight at 4 °C. Ethanol was removed by centrifugation and cellular pellets washed with HBSS-Glc. Finally, cells were resuspended in 500 μL HBSS-Glc with 20 μg/mL propidium iodide (PI) and 3 mg/mL Ribonuclease A, and incubated in darkness at r. t. for 30 min. PI fluorescence was measured in a Coulter XL EPICS flow cytometer (Beckman Coulter, Nyon, Switzerland) (λ_EXC_ = 488 nm, λ_EM_ = 620 nm) [50].

Each assay was repeated at least twice. Untreated cells were used as control for a standard cell cycle histogram, and Miltefosine (hexadecylphosphocholine) (15 μM) was used as control for an apoptosis-inducing respiratory inhibitor [51].

## 3. Results and Discussion

### 3.1. Computational Analysis of Leishmania GSK-3 Structure

In an initial step, identification of druggable cavities in the selected target was carried out to identify small-molecule ligands. The initial sequence alignment analysis of the human and the parasite enzyme (Supplementary Materials, Figure S1) showed that, despite a reasonable degree of dissimilarity between the *Leishmania* GSK-3 and its human orthologue, the identity in the ATP binding pocket is highly preserved, including the conserved Cys199 (Cys169 in LmjGSK-3s), critical for the binding of covalent inhibitors [52,53]. Remarkably, two residues differed in this region, Asp133 and Leu131; in the human enzyme, these were replaced, respectively, by Glu101 and Met100 in LmjGSK-3s, leading to a slight increase in the bulkiness of these residues located at the hinge region in the ATP-binding site. Based on this, and with the goal of identifying potential binding cavities in the parasite protein, we first proceeded to the surface analysis studies using the crystal structure available from LmjGSK-3s [31]. For this purpose, protein-druggable sites and their potential fragment-binding sites for the protein kinase inhibitors (PKI) of each pocket were uncovered using the free geometry-based algorithm Fpocket [43] and MDpocket [44], as well as by the prediction of fragment hotspot maps [45]. 

**Figure 1:**
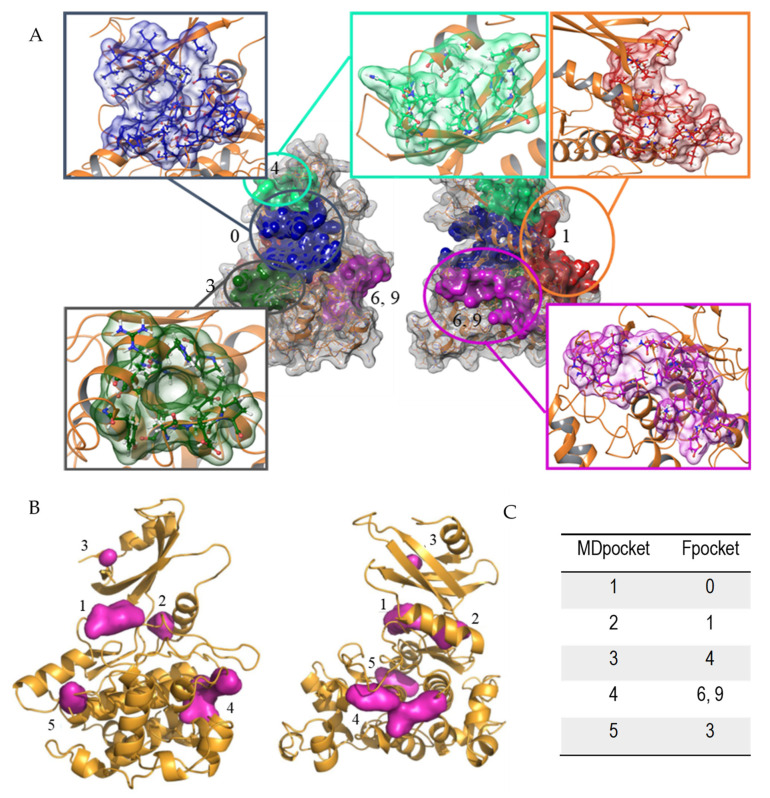
**Pockets found in LmjGSK-3s structure**. (**A**) Principal cavities found by Fpocket software front (**left**) and back view (**right**). (**B**) Conserved pockets in the different GSK-3 structures analysed by MDpocket, front (**left**) and back view (**right**). (**C**) Relationship between the respective numbering of MDpocket vs. Fpocket.

Fpocket identified 16 potential cavities in the structure of the protein, that were ranked by score and volume based on the clustering of alpha spheres and Voroni tessellation, according to alpha sphere density, polarity, or hydrophobic density [43]. All of them underwent further visual inspection. The results pinpointed significant facts. Most of the cavities previously described for hGSK-3β [53] appeared also in the LmjGSK-3s protein. The top-scoring pockets were the ATP-binding site, with its characteristic hinge region connecting the *C*- and *N*-lobes, the substrate-binding pocket next to the ATP-binding site, as well as several allosteric regions. Moreover, most of the residues that form the ATP-binding site (Pocket 0, depicted in Figure 1A) and the substrate pocket were highly conserved, while allosteric pockets showed higher differences respect to their homologues of the human enzyme. In order to analyse the conserved pockets in the different GSK-3 structures, the 56 structures available in the PDB [54] at the time of the study, including that from *Leishmania*, were retrieved. These structures, mostly from the human enzyme, were analysed by MDpocket [44]. The purpose is to identify potential differences between the conserved pockets of the structures, to be later compared with those of the *Leishmania* enzyme. 

From these analyses, those pockets with high score in Fpocket were interrelated with those from MDpocket analysis in Figure 1A. According to Figure 1B, the ATP-binding site was by far the pocket with the highest conservation, named Pocket 0, in agreement with the aforementioned sequence comparison. In addition, four additional pockets were partially preserved in the different structures, but with higher divergences in volume and shape. Figure 1C shows the correlation between the top ranked cavities of LmjGSK-3s from Fpocket analysis with those pockets found in MDpocket.

**Figure 2:**
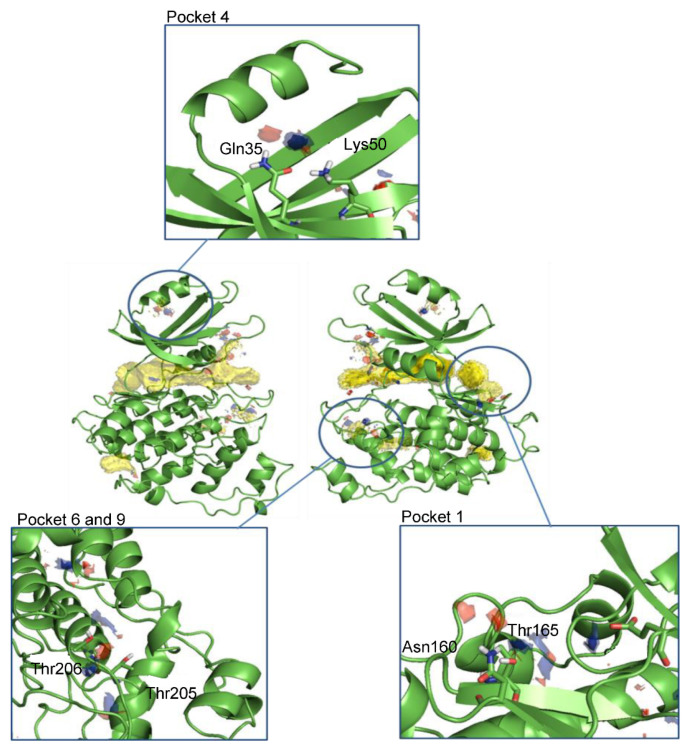
**Front and back view of the hotspots maps in the entire LmjGSK-3s protein and a detailed view of the pockets selected for virtual screening purposes**. Apolar hotspots were hidden in the detailed pictures for visualization purposes.

After the analysis of the surface and clustering of all cavities in the search for potential allosteric pockets, Hotspots maps studies [45] identified the top pockets areas where key interactions driving ligand–protein binding might be established. As expected, the ATP-binding site accumulated most of the total hotspot maps. In addition, other areas within the pockets found in the cavity search analysis were spotted as potential sites for ligand binding by chemical probes (Figure 2).

Altogether, four allosteric pockets, in addition to the ATP-binding site, were identified by this approach. 

### 3.2. Searching for Novel Inhibitors Using Virtual Screening

The survey for new and highly selective active structures of *Leishmania* GSK-3 within the MBC chemical library [47] was performed by virtual screening on the pockets found in the LmjGSK-3s. A set of 24 compounds chemically diverse, were prioritised for biological evaluation according to the Glide XP Score and the hotspots maps calculations. 

The inhibition of the enzymatic activity of the purified LdGSK-3s by the 24 selected compounds, that represented a variety of chemical scaffolds, was evaluated using Kinase Glo^®^ (Promega Biotech Ibérica, Alcobendas, Spain) (Supplementary Material, Table S1) as previously described [34]. Out of the 24 compounds, only naphthoquinone MBC-10 showed a moderate inhibition of LdGSK-3s (45.7 ± 7.6% inhibition at 10 µM) (Table 1). This result prompted us to evaluate additional molecules related to it within our in-house chemical library [47]. A set of related structures bearing a benzo- or a naphthoquinone core with different substituent patterns were prioritised and subsequently tested. The quinone MBC-132, with a carbamate group in position 2 and a chlorine atom in position 3 has an IC_50_ of 2.5 ± 0.1 µM on LdGSK-3s inhibition, with 4-fold improvement over MBC-10 (Table 1). Then, the leishmanicidal activity of these two molecules were subsequently assessed on axenic promastigotes and amastigotes. Both molecules resulted as appealing candidates under the phenotypic screening. MBC-10 showed IC_50_s of 10.5 ± 1.2 µM and 11.2 ± 2.5 µM on promastigotes and axenic amastigotes, respectively, in the same trend as their inhibitory enzymatic activity. MBC-132 showed IC_50_s in the low micromolar and sub-micromolar range: 1.51 ± 0.02 µM and 0.51 ± 0.01 µM for promastigotes and axenic amastigotes, respectively (Table 1), improving by 10-fold the MBC-10 values, with a selectivity index of 5.1 on mouse peritoneal macrophages. 

Noteworthy, quinone scaffolds such as *p*-benzoquinone or naphthoquinone [55,56] were considered as privileged structure in drug discovery [57]. Moreover, several quinone derivatives are drugs approved for different pathologies [58]. However, to the best of our knowledge, inhibition of GSK-3 enzymes by quinones has not been reported yet, although it was for other PKs through binding to the ATP binding pocket, such as anthraquinones for human PIM1 kinase [59]. Other modes of PK inhibition by quinones include binding of naphtho(hydro)quinone into the allosteric sites of the p21-activated kinase PAK [60], or the covalent allosteric inhibition of Akt by 5,7-dimethoxy-1,4-phenanthrenequinone [61]. 

The biological properties of quinones are mostly based either on their extensive redox metabolism, frequently inducing ROS production, or as Michael acceptors to form adducts with highly nucleophilic residues [62]. The chemical properties, and hence their potential as therapeutics, are modulated by the substituents of the scaffold [63,64]. Therefore, following a ligand-based drug design strategy a new set of derivatives from MBC-132 were synthesized to explore structure–activity relationships (SAR).

### 3.3. Design, Synthesis, Biological Evaluation, and SAR Analysis of a Second Generation of Naphthoquinone Derivatives 

**Figure 3:**
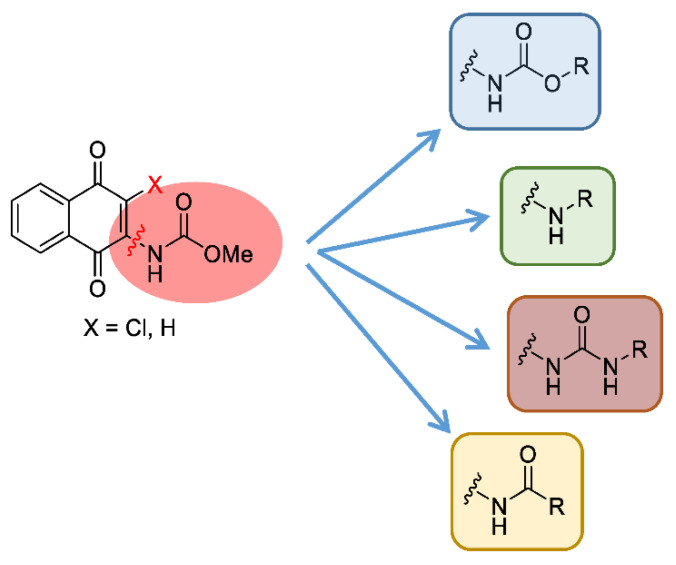
**Chemical modifications proposed to explore the chemical space of the naphthoquinone scaffold (X accounts either for Cl or H)**.

MBC-132 improved substantially the LdGSK-3s inhibition and its associated leishmanicidal values with respect to the initial compound MBC-10. To further optimize this new hit in terms of activity and selectivity, a medicinal chemistry approach was developed to explore the chemical space of the naphthoquinone scaffold. For this, a dedicated synthesis of a new generation of naphthoquinones was carried out with a double aim; to assess the role of the chemical nature of the substituent at position 2, and to define the importance of the chlorine at position 3. The first objective was tackled by the synthesis of a subset of compounds that maintained the chlorine atom at position 3, but with different functional groups at position 2, such as amines, urea, or amides, as well as the initial carbamate itself. For the second objective, a new series of naphthoquinone derivatives compounds without the chlorine in position 3 was synthesized to determine its influence on the activity (Figure 3).

**Scheme 1:**
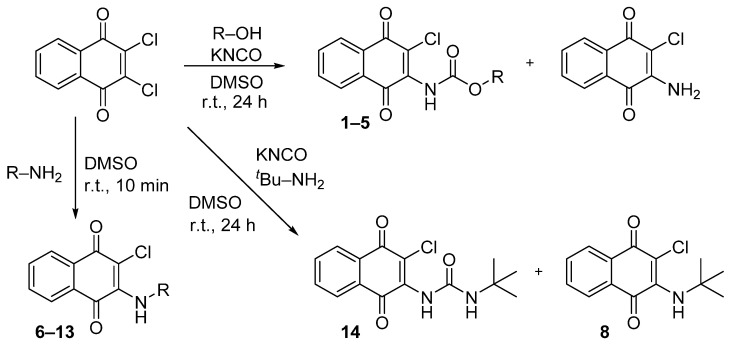
Synthesis of naphthoquinone derivatives with a carbamate moiety (**1–5**), with an amine group (**6–13**) and of the urea derivative **14**. (r.t., room temperature).

A previously described procedure [35] was followed to synthesize a series of carbamate derivatives **1–5** generated with a moderate yield, by the use of the corresponding aliphatic and aromatic alcohols. The fast kinetics of this reaction minimized the hydrolysis of the intermediate isocyanate, but the formation of the 2-amino-3-chloro-1,4-naphthoquinone was occasionally observed, as previously reported [35]. Secondly, the influence of the carbamate moiety in the biological activity was explored with a new set of naphthoquinone derivatives in which the carbamate was replaced either by an amine group, or by a urea moiety at the same position. In the first case, derivatives (**6–13**) were obtained by reaction of the 2,3-dichloro-1,4-naphthoquinone with different aliphatic and aromatic amines in anhydrous DMSO at r. t. with good yields (Scheme 1). The replacement of carbamate by urea followed a similar chemical strategy employed for the carbamate synthesis, but with the use of primary amines differently substituted, either aliphatic or aromatic. Nevertheless, a final urea derivative was obtained only for *tert*-butylamine (**14**). For the other cases, instead of the expected urea formation, the direct addition of the amine occurred due to the low nucleophilic properties of the potassium cyanate compared with the respective amine. 

Finally, the role of the chlorine atom at position 3 in the biological activity of this chemical class of compounds was assessed by synthesis of 1,4-naphthoquinone derivatives devoid of this substituent.

**Scheme 2:**
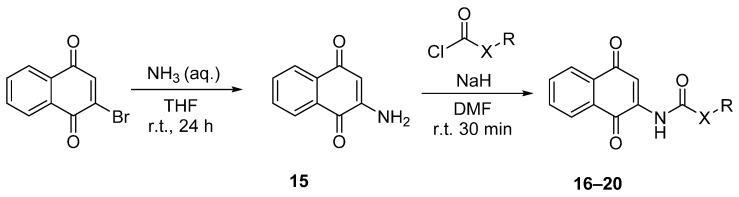
Synthesis of 2-carbamate-1,4-naphthoquinone derivatives **16–17** and 2-amide-1,4-naphthoquinone derivatives **18–20**. (aq., aqueous; DMF, *N,N*-dimethylformamide; r.t., room temperature; THF, tetrahydrofuran).

For this, amide and carbamate naphthoquinone derivatives were synthesized from 2-amino-1,4-naphthoquinone as the starting material under a two-step protocol. In the first step, 2-amino-1,4-naphthoquinone (**15**) was obtained by treatment of 2-bromo-1,4-naphthoquinone with aqueous ammonia, 32% [65]. The moderate yields and the formation of secondary products were substantially improved by the replacement of the aqueous ammonia as solvent by THF (r. t., 24 h) (Scheme 2). In the second step, the reaction of the quinone **15** with the respective chloroformate or acid halide led to the formation of the corresponding carbamates **16–17** or amide derivatives **18–20** [66]. The nucleophilic substitution was performed in the presence of sodium hydride, obtaining the final compounds with moderate yields. The identification and characterization of the compounds were included within the Materials and Methods Section.

In the next step, inhibition of the LdGSK-3s was evaluated for the 19 newly synthesized quinones (compounds **1–14** and **16–20**, Table 2). As with the parent compound MBC-132, its carbamate derivatives (**1–5**) maintained their respective IC_50_s at the low micromolar range. However, a 4-fold increase in IC_50_s (>10 μM) occurred when either the carbamate moiety was replaced by urea, amide, or amine, or the chlorine in position 3 was absent. 

In a step ahead, the leishmanicidal activity of the compounds on *L. pifanoi* axenic amastigotes and *L. infantum* promastigotes was evaluated. Axenic *Leishmania* amastigotes afford the appraisal of the leishmanicidal activity without the constrains imposed on the access of the compounds to intracellular amastigotes within the parasitophorous vacuole, as well as to preclude the interference of the compounds via host–parasite interface in the final activity. For that, the axenic line of *L. pifanoi* amastigotes belonging to the mexicana complex was chosen, as in this species the infectivity, antigenicity, and metabolic identity between axenic and intracellular amastigotes have been consistently validated [67].

The IC_50_s for compounds **1–5** on both axenic forms of the parasite fell within the low micromolar and sub-micromolar ranges. Compounds with an aliphatic substitution of the carbamate (**1** and **2**), improved slightly the activity of the MBC-132 on the amastigote, while on promastigote, values were rather similar. The replacement of an aliphatic carbamate (**1–2**) by an aromatic one (**3–5**) produced a dissimilar effect on the leishmanicidal activity; whereas on promastigotes IC_50s_ were maintained at low micromolar values, regardless of the aromatic or aliphatic nature of the substituents, on amastigotes, the IC_50_s were higher for aromatic than for aliphatic derivatives. The selectivity index of **1** and **2** increased 2- and 4-fold with respect to MBC-132 (Table 2), also driven by their lower toxicity on peritoneal macrophages. Compound **1**, the carbamate with an ethyl substituent, resulted with the highest selectivity index (SI = 20.6). In contrast, the slightly higher toxicity on macrophages, and the lower activity on amastigotes, decreased the SI for **3–5** below MBC-132.

Replacement of the carbamate by an amine at position 2 (compounds **6–13**), plummeted IC_50_ values for both forms of the parasite, even for aliphatic substituents (**6–8**). These compounds showed, in vitro, a nil inhibition of LdGSK-3s in agreement with their poor leishmanicidal activity. The substitution of the carbamate by urea (compound **14**) led to an activity on promastigotes comparable to the initial hit MBC-132; although, their higher cytotoxicity on macrophages and lower activity on amastigotes ruined their selectivity index. 

The elimination of the chlorine at position 3 (compounds **16–20**) maintained the leishmanicidal activity of the reference MBC-132. However, they showed a lower selectivity index because of their higher toxicity on macrophages. 

Altogether, the presence of the carbonyl moiety (carbamate and urea derivatives) is essential; its replacement by amine led to derivatives inactive or poorly active (**6–13**). The leishmanicidal activity was not severely modified by the absence of the chlorine atom at position 3 (**16–20**), but impaired their selectivity index below 3. 

**Figure 4:**
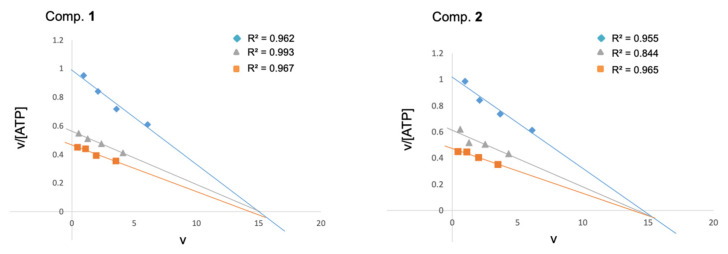
**Eadie–Scatchard plots of the inhibition of LdGSK-3s by quinone derivatives 1 and 2**. ATP concentrations in the reaction mixture ranged from 1 to 10 μM. Inhibitor concentrations used were 0 (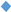), 2.5 (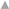) and 5 (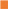) μM, respectively. The phospho-glycogen synthase peptide-2 (GS2) was used as a substrate at a fixed concentration of 25 μM. Each kinetic point was made by duplicate, and represented as the mean of two independent experiments.

The structural modifications on the quinone scaffold led to active derivatives whose substitution pattern widely differed from the initial hit MBC-10. In order to decipher the mechanism of inhibition, experimental enzymatic kinetic studies were performed. Two of the most active LdGSK-3s inhibitors, quinones **1** and **2**, were chosen to study their competition with ATP. Kinetic experiments with variation of the concentration of either ATP (from 1 to 10 μM) or the inhibitors **1** and **2** (from 2.5 to 5 μM) were performed. An Eadie–Scatchard plot (v vs. v/[ATP]) of the data is depicted in Figure 4. The intercept of the lines just below the x-axis endorsed that both compounds act as ATP competitive inhibitors. This mode of inhibition could be presumably extrapolated to similar compounds as MBC-132 or **3–5**.

Noteworthy, the best LdGSK-3s inhibitors (MBC-132, **1–3**) showed a poor inhibition of hGSK-3β inhibition (at 10 μM the percentages of inhibition were 19, 30, 25, and 19%, respectively). To the best of our knowledge, these are the first LdGSK-3s inhibitors with modest affinity for hGSK-3β reported to date, with compounds **1** (SI = 20.6) and **2** (SI = 11.2) being the lead molecules of these series of naphthoquinone in the phenotypic assay, as they showed the highest SI values. 

In this regard, the specific inhibition of LdGSK-3s over hGSK-3β has a special relevance in leishmaniasis. The resolution of the disease relies on the induction of a proinflammatory process, where activation of the macrophages and elimination of intracellular parasites ensued. The pleiotropic function of GSK-3β in macrophages was recently reported [68], as well as the ambiguous and complex modulation of inflammation by GSK-3β [69,70]. Works addressing the role of hGSK-3β on leishmaniasis support the beneficial activity of this enzyme for parasite elimination [71,72]. Consequently, these LdGSK-3s inhibitors, themselves lethal to the parasites, partially spare the inhibition of hGSK-3β, with a presumed synergic leishmanicidal effect, caused by the activity of the human enzyme, while that of the parasite was selectively inhibited. 

### 3.4. Leishmanicidal Activity on Intracellular Amastigotes of LdGSK-3s Quinone Inhibitors

The assay of drugs on intracellular parasites is a model closer to the natural infection, due to the inclusion of traffic and accumulation of the drug into the parasitophorous vacuole, as well as feasible effects at the host–parasite interface. Consequently, the six quinones with inhibitory activity on LdGSK-3s and leishmanicidal activity on axenic amastigotes (compounds MBC-132 and **1–5**) were tested on macrophages infected with *L. pifanoi* amastigotes.

**Figure 5:**
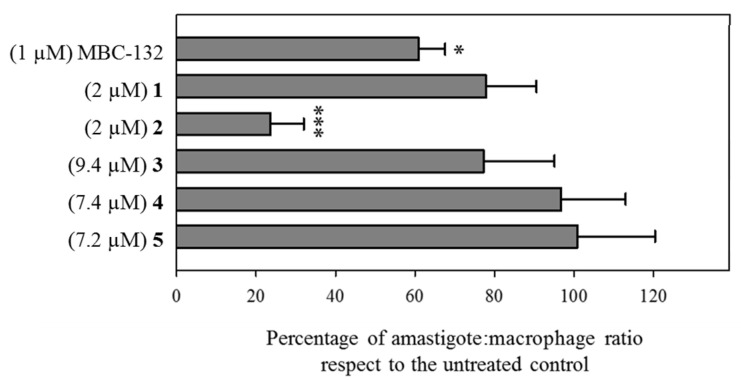
**Variation of the parasite load of BALB/c murine peritoneal macrophages infected with mCherry-*L. pifanoi* axenic amastigotes after treatment with selected LdGSK-3s inhibitors**. Macrophages were infected with a 3:1 parasite:macrophage ratio for 4 h. Afterwards, infection was allowed to progress for 24 h. Then, macrophages were challenged with the corresponding concentration of each compound for 24 h, and changes in the parasite:macrophage ratio was obtained by fluorescence microscopy. Compounds were added from stock solutions in DMSO. The same final concentration of DMSO (0.25% *v*/*v*) was present in all the samples, including control untreated infected macrophages). Data were referred as the ratio percentage of treated vs. untreated macrophages. Student’s *t*-test (*: *p* < 0.05, ***: *p* < 0.001).

**Figure 6:**
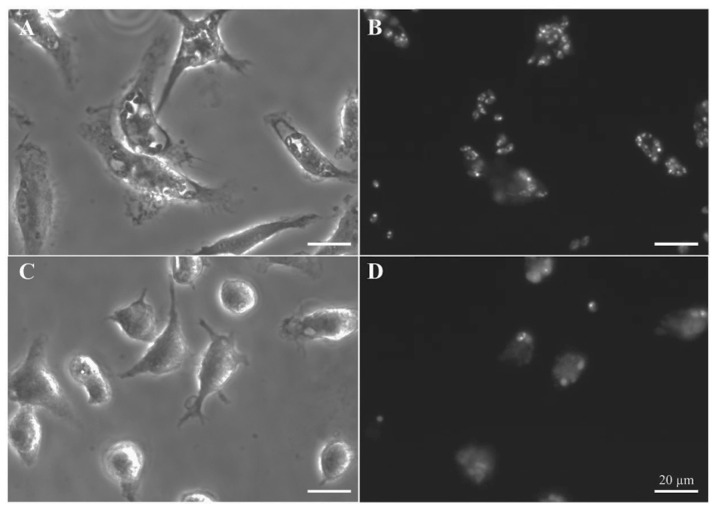
**Leishmanicidal activity of quinone 2 in intracellular *L. pifanoi* amastigotes**. Representative fields of BALB/c murine peritoneal macrophages infected with mCherry–*L. pifanoi* axenic amastigotes. Macrophages were infected with a 3:1 parasite:macrophage ratio for 4 h. Afterwards, infection was allowed to progress for 24 h. Then, macrophages were challenged with the corresponding concentration of each compound for 24 h. The final percentage for DMSO in the incubation medium was 0.25% *v*/*v*, including control, untreated macrophages. (**A**,**B**) Untreated macrophages. (**C**,**D**) Infected macrophages treated with quinone **2** at 2 μM (5-fold its IC_50_ in axenic amastigotes). (**A**,**C**) Phase microscopy. (**B**,**D**) Fluorescence microscopy (540–580 nm excitation filter). Magnification bar: 20 μm.

Compounds MBC-132 and **2** showed the higher decrease in parasite load (Figure 5). Quinone **2** at 2 μM induced the highest decrease (76.3%), whereas quinone MBC-132 at 1 μM caused a decrease of 39.1%. In all, we surmise that the loss of effectiveness in intracellular infections for 2 of the 6 quinones relative to axenic amastigotes is likely due to a faulty access of the quinone to the intracellular parasite. Figure 6 showed representative images of infections treated with compound **2**. 

### 3.5. Energy Metabolism of Leishmania as an Off-Target Effect of LdGSK-3s Inhibitors

The redox chemistry of quinones makes them suitable candidates to mimic ubiquinone, a natural quinone working as electron carrier within the respiratory chain, thus with a potential interference with the electron transport of the respiratory chain of trypanosomatids, including *Leishmania* [73,74]. Therefore, appraisal of the effects of the quinones described here on the energy metabolism of promastigotes was undertaken.

#### 3.5.1. Inhibition of the Electrochemical Potential of the Leishmania Mitochondrion (Δ*Ψ_m_*)

In *Leishmania*, especially in the promastigote, oxidative phosphorylation is the main source for ATP biosynthesis [75], dependent on the maintenance of the electrochemical potential (Δ*Ψ_m_*) created by the respiratory chain.

**Figure 7:**
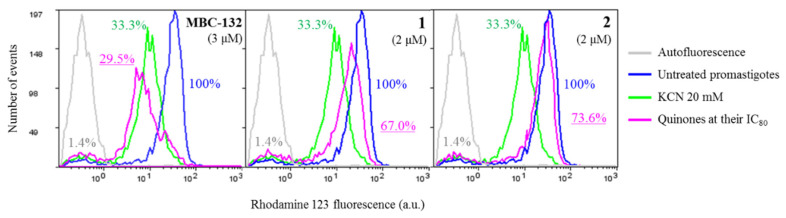
**Variation of intracellular accumulation of Rhodamine 123 (Rh123) in *L. donovani* promastigotes by selected quinones**. Parasites were incubated with the compounds at their IC_80_ for 4 h prior to Rhodamine 123 uptake, as described in the Materials and Methods Section. Rhodamine 123 accumulation was measured by flow cytometry (λ_EXC_ = 488 nm, λ_EM_ = 520 nm). Fluorescence values were expressed as the percentage with respect to the untreated control. Incubation with 20 mM KCN for 40 min was used as positive control for mitochondrial membrane depolarization.

Variation of the Δ*Ψ_m_* of *Leishmania* parasites were monitored through the preferential accumulation of rhodamine 123 (Rh123) within the mitochondrion [50], driven by the Nernst equation. Quinones MBC-132, **1,** and **2** were incubated for 4 h at their respective IC_80_s on promastigotes prior to Rh123 accumulation. A 40 min incubation with 20 mM KCN was used as a control inhibitor of oxidative phosphorylation (Figure 7). Quinone MBC-132 (3 μM) induced the highest decrease in Rh123 accumulation, similar to the KCN-treated control (ca 70%). Quinones **1** and **2** showed similar effects at 2 μM, with decrease of 33.0 and 26.4% Rh123 fluorescence, respectively, supporting an inhibitory activity on the oxidative phosphorylation of *L. donovani*, also described for other quinones in *Leishmania* [76,77,78].

#### 3.5.2. Inhibition of Oxygen Consumption

**Figure 8:**
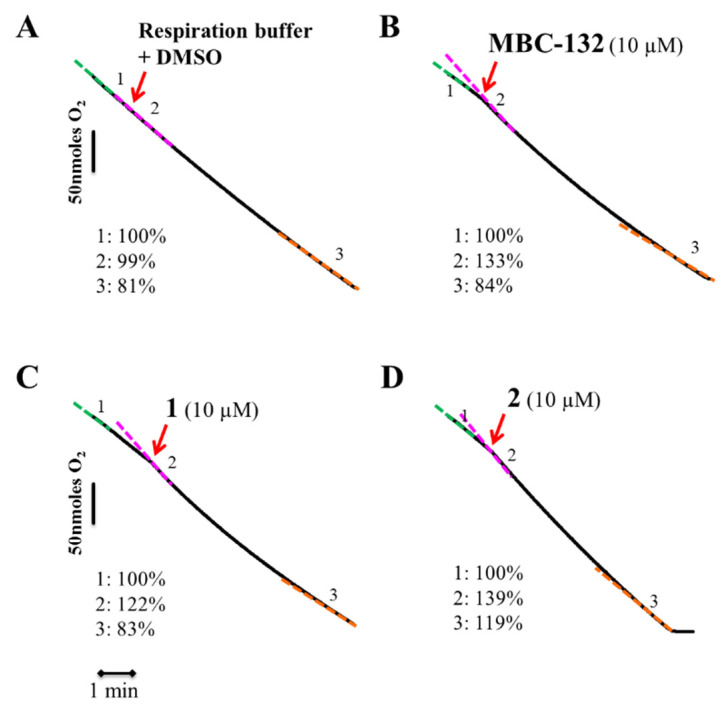
**Oxygen consumption rates of *L. donovani* parasites with quinones MBC-132, 1, and 2**. Parasites were resuspended in respiration buffer (1 × 10^8^ cells/mL) in a Clark’s oxygen electrode. After stabilization of the respiration rate, compounds were added from a stock at 100-fold final concentration. Variation in the oxygen consumption rate were recorded for 8–10 min. Red arrow—the addition of the respective compound and their respective code number and concentration used in bold black over the upper part of the trace. (**A**) Control parasites treated with respiration buffer plus DMSO at same final concentration as the quinones. (**B**) MBC-132 at 3.3-fold its IC_80_. (**C**,**D**) compounds **1** and **2**, respectively, at a concentration 5-fold IC_80_s. Respiration rates were expressed as the percentage of the O_2_ consumption rate respect to their basal rate, before addition of the compound. The different colours of the dotted lines highlight the different slopes, with respective percentage values appear at the lower left corner of each panel. The final concentration of DMSO in all the samples was 0.1% *v*/*v*.

To confirm the dysfunction of the respiratory chain achieved by the different compounds, the inhibition of respiration was assessed by polarographic methods using a Clark oxygen electrode. The cellular density required for this technique is five times higher than that used for the other bioenergetic assays. Consequently, quinones MBC-132, **1**, and **2** were assayed at 3.3–5-fold their IC_80_ (10 μM) (Figure 8) for inhibition of the respiration of *L. donovani* promastigotes. Quinones MBC-132, **1** and **2** induced an initial acceleration of the O_2_ consumption rates in the *L. donovani* promastigotes, followed by a deceleration, which, only for quinone **2**, was still higher than the initial respiration rate, prior to quinone addition. The same process of deceleration, although in a lesser extent occurred in control parasites (Figure 8A), due to the slight deviation underwent by the electrode at low oxygen concentrations, even in the absence of cells using chemical reducing agents. Thus, a dual quinone effect of the quinones with a poor and late inhibition of the oxygen consumption rate may be reasonably discarded. 

#### 3.5.3. Induction of Programmed Cell Death in *L. donovani* Promastigotes

The role of mitochondrion is mandatory for the intrinsic pathway of the programmed cell death, the only pathway available for trypanosomatids [79]. The induction of apoptotic-like processes in promastigotes was evaluated for quinones MBC-132, **1,** and **2** at their respective IC_80_s on *L. donovani* promastigotes, selected as inducers of mitochondrial depolarization, and evaluated by the induction of subG_0_/G_1_ population in flow cytometry studies by DNA staining with propidium iodide (PI). Miltefosine at 15 μM was used as a positive control for this process [50]. 

**Figure 9:**
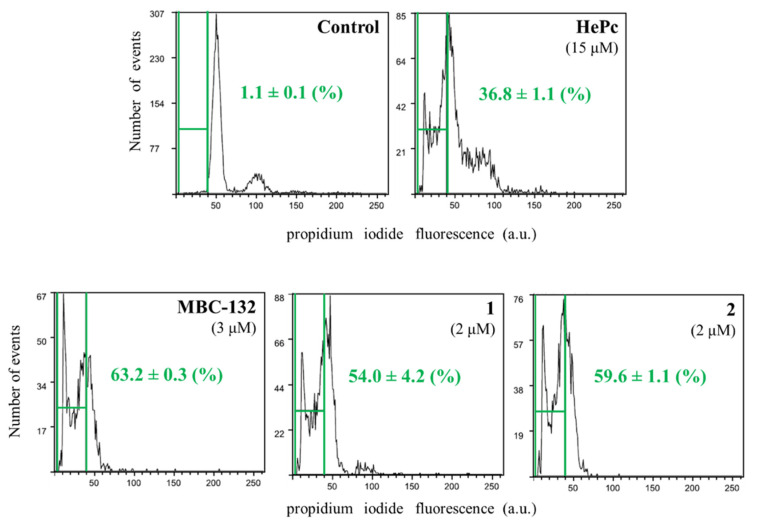
**Cell cycle of *L. donovani* promastigotes treated with quinones MBC-132, 1, and 2 at their IC_80_**. Parasites were incubated with the corresponding drug for 72 h, followed by EtOH permeabilization, incubated with 3 mg/mL Ribonuclease A and stained with 20 μg/mL of propidium iodide (PI) before cytofluorometric analysis. (λ_EXC_ = 488 nm, λ_EM_ = 620 nm). The percentage of subG_0_/G_1_ population (enclosed within green bars) stands for degraded chromatin, associated with the apoptotic-like process. Miltefosine (hexadecylphosphocholine, HePc) at 15 μM was added as a control for induction of apoptosis.

As shown in Figure 9, the three selected quinones induced programmed cell death in *L.donovani* promastigotes, with increase in the subG_0_/G_1_ population in treated promastigotes. Therefore, the three naphthoquinones studied induced a bioenergetic collapse in the promastigote of *Leishmania* by targeting the respiratory chain of the parasite leading to apoptosis, thus adding a new off-target mechanism of action to these LdGSK-3s inhibitors.

## 4. Conclusions

In all, quinones have been added as a new and appealing template for leishmanicidal LdGSK-3s inhibitors, active on two quite different *Leishmani*a species, from a clinical perspective. These naphthoquinones showed a multitarget leishmanicidal mechanism that may also encompass the respiratory chain, as it is the case here. Far from being detrimental for its pharmacological development, this fact presents an immediate advantage to curtail resistance induction by target mutation. As such, multitarget drugs have been hailed as relevant approach for neglected tropical diseases [80] with miltefosine as a paradigm [81], in tune with a current trend that advocate for a polypharmacology approach for neglected diseases. 

## Figures and Tables

**Table 1:** Enzymatic and biological characterization of MBC-10 and MBC-132 prioritised after the virtual screening and subsequent experimental validation ^a^.

Comp.	Chemical Structure	LdGSK-3s ^b^ IC_50_ (μM)	*L. donovani* Promastigotes IC_50_ (μM)	*L. pifanoi* Axenic Amastigotes IC_50_ (μM)	MPM ^c^ IC_50_ (μM)	Selectivity Index (SI) ^d^
**MBC-10**	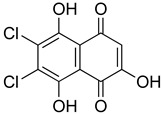	~10 ^e^	10.5 ± 1.2	11.2 ± 2.5	ND	ND
**MBC-132**	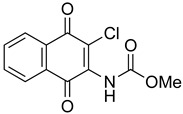	2.5 ± 0.1	1.51 ± 0.02	0.51 ± 0.01	2.6 ± 0.0	5.1

^a^ IC_50_—50% inhibitory concentration. ^b^ Indirubin-3′-monoxime-5-sulphonic acid was used as reference of the assay: IC_50_ (LdGSK-3) = 2.4 ± 0.2 μM. ^c^ MPM—mouse peritoneal macrophages. ^d^ SI—selectivity index (IC_50_ MPM/IC_50_ axenic amastigotes). ^e^ MBC-10 at 10 μM causes 45.7 ± 7.6% inhibition of LdGSK-3s activity.

**Table 2:** Enzymatic and biological characterization of MBC-132 derivatives ^a^.

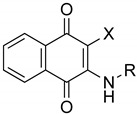							
Comp.	R	X	LdGSK-3s ^b^ IC_50_ (μM)	*L. infantum* Promastigotes IC_50_ (μM)	*L. pifanoi* Axenic Amastigotes IC_50_ (μM)	MPM ^c^ IC_50_ (μM)	Selectivity Index (SI) ^d^
**MBC-132**	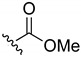	Cl	2.5 ± 0.1	1.51 ± 0.02	0.51 ± 0.04	2.6 ± 0.0	5.1
**1**	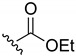	Cl	3.7 ± 0.3	1.54 ± 0.01	0.15 ± 0.40	3.1 ± 0.1	20.6
**2**	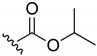	Cl	2.5 ± 0.2	1.47 ± 0.02	0.4 ± 0.2	4.51 ± 0.30	11.2
**3**	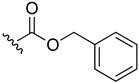	Cl	2.8 ± 0.1	4.9 ± 0.2	4.7 ± 0.8	9.8 ± 0.2	2.1
**4**	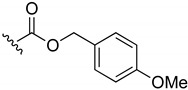	Cl	4.1 ± 0.1	2.9 ± 0.2	3.7 ± 1.1	7.4 ± 0.8	2.0
**5**	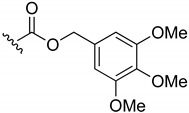	Cl	4.5 ± 0.6	3.8 ± 1.9	3.6 ± 0.6	8.1 ± 1.0	2.3
**6**	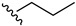	Cl	>10	19.1 ± 1.2	>25	-	-
**7**	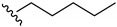	Cl	>10	16.9 ± 0.4	>25	-	-
**8**	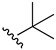	Cl	>10	19.8 ± 3.6	17.8 ± 1.1	17.0 ± 0.3	1.0
**9**	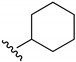	Cl	>10	>25	>25	-	-
**10**	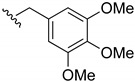	Cl	>10	>50	>50	-	-
**11**	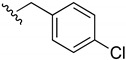	Cl	>10	>25	>25	-	-
**12**	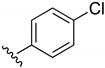	Cl	>10	>50	>50	-	-
**13**	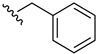	Cl	>10	>25	>25	-	-
**14**	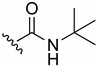	Cl	>10	1.4 ± 0.4	2.1 ± 0.6	1.3 ± 0.3	0.6
**16**	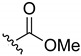	H	>10	4.1 ± 0.8	1.7 ± 0.2	3.8 ± 0.3	2.2
**17**	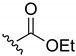	H	>10	4.5 ± 0.7	1.7 ± 0.1	4.2 ± 0.2	2.5
**18**	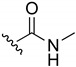	H	>10	4.5 ± 0.5	1.2 ± 0.1	4.0 ± 0.2	3.3
**19**	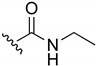	H	>10	5.9 ± 1.8	1.8 ± 0.3	4.3 ± 0.3	2.4
**20**	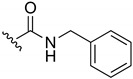	H	>10	7.1 ± 2.1	3.2 ± 0.1	7.5 ± 0.6	2.3

^a^ IC_50_—50% inhibitory concentration. ^b^ Indirubin-3′-monoxime-5-sulphonic acid was used as reference of the assay: IC_50_ (LdGSK-3) = 2.4 ± 0.2 μM. ^c^ MPM—mouse peritoneal macrophages. ^d^ SI—selectivity index (IC_50_ MPM/IC_50_ axenic amastigotes).

## Data Availability

The data presented in this study are available in the article and Supplementary Materials and upon request from the corresponding authors.

## Supplementary Materials

The following supporting information can be downloaded at: https://www.mdpi.com/article/10.3390/biomedicines10051136/s1, Figure S1: Sequence alignment of the GSK-3 structures of *Leishmania major* (Q4QE15) and *Homo sapiens* (P49841), Table S1: Chemical structures of the 24 compounds from the MBC library selected by virtual screening and their biological evaluation.

## Abbreviations

AcOEt—ethyl acetate; ASTM—American Society for Testing and Materials; ATP—adenosine triphosphate; CK1.2—casein kinase 1.2; ^13^C NMR—^13^C nuclear magnetic resonance; CRISPR-Cas9—clustered regularly interspaced short palindromic repeats-associated protein 9; 2D—two-dimensional; 3D—three-dimensional; d—doublet; dd—doublet of doublets; DEAE—diethylaminoethyl; DMF—*N,N*-dimethylformamide; DMSO—dimethyl sulfoxide; DNA—deoxyribonucleic acid; EGTA—ethylene glycol tetraacetic acid; Equiv.—equivalents; ESI—electrospray ionization; FDA—U. S. Food and Drug Administration; GSK-3—glycogen synthase kinase 3; GSK-3s—glycogen synthase kinase 3 short form; GS2—phospho-glycogen synthase peptide-2; HePc—hexadecylphosphocholine (miltefosine); HEPES—4-(2-hydroxyethyl)-1-piperazine ethanesulfonic acid; Hex—hexane; HIFCS—heat-inactivated foetal calf serum; hGSK-3β—human GSK-3β; ^1^H NMR—^1^H nuclear magnetic resonance; HRMS—high-resolution mass spectrometry; IC_x_—inhibitory concentration that decreases enzymatic activity or parasite growth by x%; *J*—coupling constant; LC—liquid chromatography; LdGSK-3s—*L. donovani* GSK-3 short form; Lit.—literature; LmjGSK-3s—*L. major* GSK-3 short form; m—multiplet; MBC—medicinal and biological chemistry; Me—methyl; MeOH—methanol; Mp—melting point; MPM—mouse peritoneal macrophages; MTT—3-(4,5-dimethylthiazol-2-yl)-2,5-diphenyltetrazolium bromide; M199—medium 199; ND—not determined; PAK—p21-activated kinases; PBS—phosphate-buffered saline; PDB—protein data bank; PI—propidium iodide; PIM1 kinase; PKs—protein kinases; PKIs—protein kinase inhibitors; q—quadruplet; QTOF—quadrupole time-of-flight; Rh123—rhodamine 123; RMSD—root mean square deviation; ROS—reactive oxygen species; RPMI 1640—Roswell Park Memorial Institute 1640 medium; r. t.—room temperature; s—singlet; SAR—structure activity relationships; sex—sextuplet; SI—selectivity index (IC_50_ MPM/IC_50_ axenic amastigotes); SP—standard precision; t—triplet; td—triplet of doublets; *^t^*Bu—*tert*-butyl; THF—tetrahydrofuran; UV—ultraviolet; XP—extra precision.

## References

[1] Burza S., Croft S.L., Boelaert M. (2018). Leishmaniasis. Lancet.

[2] Booth M. (2018). Climate change and the neglected tropical diseases. Adv. Parasitol..

[3] Alves F., Bilbe G., Blesson S., Goyal V., Monnerat S., Mowbray C., Ouattara G.M., Pécoul B., Rijal S., Rode J. (2018). Recent development of visceral leishmaniasis treatments: Successes, pitfalls, and perspectives. Clin. Microbiol. Rev..

[4] Ponte-Sucre A., Gamarro F., Dujardin J.C., Barrett M.P., López-Vélez R., García-Hernández R., Pountain A.W., Mwenechanya R., Papadopoulou B. (2017). Drug resistance and treatment failure in leishmaniasis: A 21st century challenge. PLoS Negl. Trop. Dis..

[5] Sundar S., Singh A. (2018). Chemotherapeutics of visceral leishmaniasis: Present and future developments. Parasitology.

[6] Braga S.S. (2019). Multi-target drugs active against leishmaniasis: A paradigm of drug repurposing. Eur. J. Med. Chem..

[7] Bhullar K.S., Lagarón N.O., McGowan E.M., Parmar I., Jha A., Hubbard B.P., Rupasinghe H.P.V. (2018). Kinase-targeted cancer therapies: Progress, challenges and future directions. Mol. Cancer.

[8] Chico L.K., Van Eldik L.J., Watterson D.M. (2009). Targeting protein kinases in central nervous system disorders. Nat. Rev. Drug Discov..

[9] Lahiry P., Torkamani A., Schork N.J., Hegele R.A. (2010). Kinase mutations in human disease: Interpreting genotype-phenotype relationships. Nat. Rev. Gen..

[10] Kini S.G., Garg V., Prasanna S., Rajappan R., Mubeen M. (2017). Protein kinases as drug targets in human and animal diseases. Curr. Enzym. Inhib..

[11] Cohen P., Cross D., Jänne P.A. (2021). Kinase drug discovery 20 years after imatinib: Progress and future directions. Nat. Rev. Drug Discov..

[12] Borba J.V.B., Silva A.C., Ramos P.I.P., Grazzia N., Miguel D.C., Muratov E.N., Furnham N., Andrade C.H. (2019). Unveiling the kinomes of Leishmania infantum and L. *braziliensis* empowers the discovery of new kinase targets and antileishmanial compounds. Comput. Struct. Biotechnol. J..

[13] Baker N., Catta-Preta C.M.C., Neish R., Sadlova J., Powell B., Alves-Ferreira E.V.C., Geoghegan V., Carnielli J.B.T., Newling K., Hughes C. (2021). Systematic functional analysis of *Leishmania* protein kinases identifies regulators of differentiation or survival. Nat. Commun..

[14] Parsons M., Worthey E.A., Ward P.N., Mottram J.C. (2005). Comparative analysis of the kinomes of three pathogenic trypanosomatids: *Leishmania major, Trypanosoma brucei* and *Trypanosoma cruzi*. BMC Genom..

[15] Garg M., Goyal N. (2015). MAPK1 of *Leishmania donovani* modulates antimony susceptibility by downregulating P-glycoprotein efflux pumps. Antimicrob. Agents Chemother..

[16] Wiese M. (1998). A mitogen-activated protein (MAP) kinase homologue of *Leishmania mexicana* is essential for parasite survival in the infected host. EMBO J..

[17] Zylbersztejn A.M.B., De Morais C.G.V., Lima A.K.C., Souza J.E.D.O., Lopes A.H., Da-Silva S.A.G., Silva-Neto M.A.C., Dutra P.M.L. (2015). CK2 secreted by *Leishmania braziliensis* mediates macrophage association invasion: A comparative study between virulent and avirulent promastigotes. BioMed Res. Int..

[18] Merritt C., Silva L.E., Tanner A.L., Stuart K., Pollastri M.P. (2014). Kinases as druggable targets in trypanosomatid protozoan parasites. Chem. Rev..

[19] Dacher M., Morales M.A., Pescher P., Leclercq O., Rachidi N., Prina E., Cayla M., Descoteaux A., Späth G.F. (2014). Probing druggability and biological function of essential proteins in *Leishmania* combining facilitated null mutant and plasmid shuffle analyses. Mol. Microbiol..

[20] Wyllie S., Thomas M., Patterson S., Crouch S., De Rycker M., Lowe R., Gresham S., Urbaniak M.D., Otto T.D., Stojanovski L. (2018). Cyclin-dependent kinase 12 is a drug target for visceral leishmaniasis. Nature.

[21] Dichiara M., Marrazzo A., Prezzavento O., Collina S., Rescifina A., Amata E. (2017). Repurposing of human kinase inhibitors in neglected protozoan diseases. ChemMedChem.

[22] Tirado-Duarte D., Marín-Villa M., Ochoa R., Blandón-Fuentes G., Soares M.J., Robledo S.M., Varela-Miranda R.E. (2018). The Akt-like kinase of *Leishmania panamensis*: As a new molecular target for drug discovery. Acta Trop..

[23] Chhajer R., Bhattacharyya A., Didwania N., Shadab M., Das N., Palit P., Vaidya T., Ali N. (2016). *Leishmania donovani* Aurora kinase: A promising therapeutic target against visceral leishmaniasis. Biochim. Biophys. Acta Gen. Subj..

[24] Ojo K.K., Gillespie J.R., Riechers A.J., Napuli A.J., Verlinde C.L., Buckner F.S., Gelb M.H., Domostoj M.M., Wells S.J., Scheer A. (2008). Glycogen synthase kinase 3 is a potential drug target for African trypanosomiasis therapy. Antimicrob. Agents Chemother..

[25] Xingi E., Smirlis D., Myrianthopoulos V., Magiatis P., Grant K.M., Meijer L., Mikros E., Skaltsounis A.L., Soteriadou K. (2009). 6-Br-5methylindirubin-3′oxime (5-Me-6-BIO) targeting the leishmanial glycogen synthase kinase-3 (GSK-3) short form affects cell-cycle progression and induces apoptosis-like death: Exploitation of GSK-3 for treating leishmaniasis. Int. J. Parasitol..

[26] Beurel E., Grieco S.F., Jope R.S. (2015). Glycogen synthase kinase-3 (GSK3): Regulation, actions, and diseases. Pharmacol. Ther..

[27] Bhavanasi D., Klein P.S. (2016). Wnt signaling in normal and malignant stem cells. Curr. Stem Cell Rep..

[28] Duda P., Akula S.M., Abrams S.L., Steelman L.S., Martelli A.M., Cocco L., Ratti S., Candido S., Libra M., Montalto G. (2020). Targeting GSK3 and associated signaling pathways involved in cancer. Cells.

[29] Hermida M.A., Dinesh Kumar J., Leslie N.R. (2017). GSK3 and its interactions with the PI3K/AKT/mTOR signalling network. Adv. Biol. Regul..

[30] Roca C., Campillo N.E. (2020). Glycogen synthase kinase 3 (GSK-3) inhibitors: A patent update (2016–2019). Expert Opin. Ther. Pat..

[31] Ojo K.K., Arakaki T.L., Napuli A.J., Inampudi K.K., Keyloun K.R., Zhang L., Hol W.G.J., Verlinde C.L.M.J., Merritt E.A., Van Voorhis W.C. (2011). Structure determination of glycogen synthase kinase-3 from Leishmania major and comparative inhibitor structure-activity relationships with *Trypanosoma brucei* GSK-3. Mol. Biochem. Parasitol..

[32] Efstathiou A., Gaboriaud-Kolar N., Smirlis D., Myrianthopoulos V., Vougogiannopoulou K., Alexandratos A., Kritsanida M., Mikros E., Soteriadou K., Skaltsounis A.L. (2014). An inhibitor-driven study for enhancing the selectivity of indirubin derivatives towards leishmanial Glycogen Synthase Kinase-3 over leishmanial cdc2-related protein kinase 3. Parasites Vectors.

[33] Efstathiou A., Smirlis D. (2021). *Leishmania* protein kinases: Important regulators of the parasite life cycle and molecular targets for treating leishmaniasis. Microorganisms.

[34] Martínez de Iturrate P., Sebastián-Pérez V., Nácher-Vázquez M., Tremper C.S., Smirlis D., Martín J., Martínez A., Campillo N.E., Rivas L., Gil C. (2020). Towards discovery of new leishmanicidal scaffolds able to inhibit *Leishmania* GSK-3. J. Enzym. Inhib. Med. Chem..

[35] Bittner S., Temtsin G., Sasson Y. (2000). Synthesis of *N*-quinonyl carbamates via 2-chloro-3-isocyanato-1, 4-naphthoquinone. Synthesis.

[36] Husu B., Kafka S., Kadunc Z., Tišler M. (1988). Amination of naphthoquinones with azidotrimethylsilane. Mon. Chem..

[37] Šali A., Blundell T.L. (1993). Comparative protein modelling by satisfaction of spatial restraints. J. Mol. Biol..

[38] (2015). Schrödinger Release 2015-4: LigPrep.

[39] Banks J.L., Beard H.S., Cao Y., Cho A.E., Damm W., Farid R., Felts A.K., Halgren T.A., Mainz D.T., Maple J.R. (2005). Integrated modeling program, applied chemical theory (IMPACT). J. Comput. Chem..

[40] Jorgensen W.L., Maxwell D.S., Tirado-Rives J. (1996). Development and testing of the OPLS all-atom force field on conformational energetics and properties of organic liquids. J. Am. Chem. Soc..

[41] Madhavi Sastry G., Adzhigirey M., Day T., Annabhimoju R., Sherman W. (2013). Protein and ligand preparation: Parameters, protocols, and influence on virtual screening enrichments. J. Comp.-Aided Mol. Des..

[42] (2015). Schrödinger Release 2015-4: Maestro.

[43] Le Guilloux V., Schmidtke P., Tuffery P. (2009). Fpocket: An open source platform for ligand pocket detection. BMC Bioinform..

[44] Schmidtke P., Bidon-Chanal A., Luque F.J., Barril X. (2011). MDpocket: Open-source cavity detection and characterization on molecular dynamics trajectories. Bioinformatics.

[45] Radoux C.J., Olsson T.S., Pitt W.R., Groom C.R., Blundell T.L. (2016). Identifying interactions that determine fragment binding at protein hotspots. J. Med. Chem..

[46] (2017). Schrödinger Release 2017-1: Glide.

[47] Sebastián-Pérez V., Roca C., Awale M., Reymond J.L., Martinez A., Gil C., Campillo N.E. (2017). Medicinal and Biological Chemistry (MBC) library: An efficient source of new hits. J. Chem. Inform. Model..

[48] Friesner R.A., Murphy R.B., Repasky M.P., Frye L.L., Greenwood J.R., Halgren T.A., Sanschagrin P.C., Mainz D.T. (2006). Extra precision glide: Docking and scoring incorporating a model of hydrophobic enclosure for protein-ligand complexes. J. Med. Chem..

[49] Palomo V., Perez D.I., Roca C., Anderson C., Rodríguez-Muela N., Perez C., Morales-Garcia J.A., Reyes J.A., Campillo N.E., Perez-Castillo A.M. (2017). Subtly modulating glycogen synthase kinase 3 β: Allosteric inhibitor development and their potential for the treatment of chronic diseases. J. Med. Chem..

[50] Luque-Ortega J.R., Rivas L. (2010). Characterization of the leishmanicidal activity of antimicrobial peptides. Methods Mol. Biol..

[51] Zuo X., Djordjevic J.T., Oei J.B., Desmarini D., Schibeci S.D., Jolliffe K.A., Sorrell T.C. (2011). Miltefosine induces apoptosis-like cell death in yeast via Cox9p in cytochrome c oxidase. Mol. Pharmacol..

[52] Domínguez J.M., Fuertes A., Orozco L., Monte-Millán M.D., Delgado E., Medina M. (2012). Evidence for irreversible inhibition of glycogen synthase kinase-3β by tideglusib. J. Biol. Chem..

[53] Palomo V., Soteras I., Perez D.I., Perez C., Gil C., Campillo N.E., Martinez A. (2011). Exploring the binding sites of glycogen synthase kinase 3. identification and characterization of allosteric modulation cavities. J. Med. Chem..

[54] Berman H.M., Battistuz T., Bhat T.N., Bluhm W.F., Bourne P.E., Burkhardt K., Feng Z., Gilliland G.L., Iype L., Jain S. (2002). The protein data bank. Acta Crystallogr. Sect. D Biol. Crystallogr..

[55] Klotz L.O., Hou X., Jacob C. (2014). 1,4-naphthoquinones: From oxidative damage to cellular and inter-cellular signaling. Molecules.

[56] Silakari P., Priyanka, Piplani P. (2020). *p*-Benzoquinone as a privileged scaffold of pharmacological significance: A review. Mini-Rev. Med. Chem..

[57] Zhang L., Zhang G., Xu S., Song Y. (2021). Recent advances of quinones as a privileged structure in drug discovery. Eur. J. Med. Chem..

[58] Ferreira V.F., de Carvalho A.S., Ferreira P.G., Lima C.G.S., da Silva F.C. (2021). Quinone-based drugs: An important class of molecules in medicinal chemistry. Med. Chem..

[59] Schroeder R.L., Goyal N., Bratton M., Townley I., Pham N.A., Tram P., Stone T., Geathers J., Nguyen K., Sridhar J. (2016). Identification of quinones as novel PIM1 kinase inhibitors. Bioorg. Med. Chem. Lett..

[60] Kim D.J., Choi C.K., Lee C.S., Park M.H., Tian X., Kim N.D., Lee K.I., Choi J.K., Ahn J.H., Shin E.Y. (2016). Small molecules that allosterically inhibit p21-activated kinase activity by binding to the regulatory p21-binding domain. Exp. Mol. Med..

[61] Chen P.J., Ko I.L., Lee C.L., Hu H.C., Chang F.R., Wu Y.C., Leu Y.L., Wu C.C., Lin C.Y., Pan C.Y. (2019). Targeting allosteric site of Akt by 5,7-dimethoxy-1,4-phenanthrenequinone suppresses neutrophilic inflammation. EBioMedicine.

[62] Bolton J.L., Dunlap T. (2017). Formation and biological targets of quinones: Cytotoxic versus cytoprotective effects. Chem. Res. Toxicol..

[63] Pinto A.V., De Castro S.L. (2009). The trypanocidal activity of naphthoquinones: A review. Molecules.

[64] Lizzi F., Veronesi G., Belluti F., Bergamini C., López-Sánchez A., Kaiser M., Brun R., Krauth-Siegel R.L., Hall D.G., Rivas L. (2012). Conjugation of quinones with natural polyamines: Toward an expanded antitrypanosomatid profile. J. Med. Chem..

[65] Yamashita M., Ueda K., Sakaguchi K., Tokuda H., Iida A. (2011). One-Pot synthesis of benzo [f] indole-4, 9-diones from 1, 4-naphthoquinones and terminal acetylenes. Chem. Pharm. Bull..

[66] Josey B.J., Inks E.S., Wen X., Chou C.J. (2013). Structure–activity relationship study of vitamin K derivatives yields highly potent neuroprotective agents. J. Med. Chem..

[67] Pan A.A., Duboise S.M., Eperon S., Rivas L., Hodgkinson V., Traub-Cseko Y., McMahon-Pratt D. (1993). Developmental life cycle of *Leishmania*—Cultivation and characterization of cultured eextracellular amastigotes. J. Eukaryot. Microbiol..

[68] Patel S., Werstuck G.H. (2021). Macrophage function and the role of GSK3. Int. J. Mol. Sci..

[69] Hoffmeister L., Diekmann M., Brand K., Huber R. (2020). GSK3: A kinase balancing promotion and resolution of inflammation. Cells.

[70] Cortés-Vieyra R., Silva-García O., Gómez-García A., Gutiérrez-Castellanos S., Álvarez-Aguilar C., Baizabal-Aguirre V.M. (2021). Glycogen synthase kinase 3β modulates the inflammatory response activated by bacteria, viruses, and parasites. Front. Immunol..

[71] Nandan D., Camargo de Oliveira C., Moeenrezakhanlou A., Lopez M., Silverman J.M., Subek J., Reiner N.E. (2012). Myeloid cell IL-10 production in response to *Leishmania* involves inactivation of glycogen synthase kinase-3β downstream of phosphatidylinositol-3 kinase. J. Immunol..

[72] Paul J., Naskar K., Chowdhury S., Chakraborti T., De T. (2014). TLR mediated GSK3β activation suppresses CREB mediated IL-10 production to induce a protective immune response against murine visceral leishmaniasis. Biochimie.

[73] Ribeiro G.A., Cunha-Júnior E.F., Pinheiro R.O., da-Silva S.A.G., Canto-Cavalheiro M.M., da Silva A.J.M., Costa P.R.R., Netto C.D., Melo R.C.N., Almeida-Amaral E.E. (2013). LQB-118, an orally active pterocarpanquinone, induces selective oxidative stress and apoptosis in *Leishmania amazonensis*. J. Antimicrob. Chemother..

[74] Prati F., Bergamini C., Molina M.T., Falchi F., Cavalli A., Kaiser M., Brun R., Fato R., Bolognesi M.L. (2015). 2-Phenoxy-1,4-naphthoquinones: From a multitarget antitrypanosomal to a potential antitumor profile. J. Med. Chem..

[75] Tielens A.G., van Hellemond J.J. (2009). Surprising variety in energy metabolism within Trypanosomatidae. Trends Parasitol..

[76] Ortiz D., Forquer I., Boitz J., Soysa R., Elya C., Fulwiler A., Nilsen A., Polley T., Riscoe M.K., Ullman B. (2016). Targeting the cytochrome bc 1 complex of *Leishmania* parasites for discovery of novel drugs. Antimicrob. Agents Chemother..

[77] Carvalho L., Luque-Ortega J.R., Manzano J.I., Castanys S., Rivas L., Gamarro F. (2010). Tafenoquine, an antiplasmodial 8-aminoquinoline, targets *Leishmania* respiratory complex III and induces apoptosis. Antimicrob. Agents Chemother..

[78] Carvalho L., Luque-Ortega J.R., López-Martín C., Castanys S., Rivas L., Gamarro F. (2011). The 8-aminoquinoline analogue sitamaquine causes oxidative stress in *Leishmania donovani* promastigotes by targeting succinate dehydrogenase. Antimicrob. Agents Chemother..

[79] Basmaciyan L., Azas N., Casanova M. (2018). Different apoptosis pathways in *Leishmania* parasites. Cell Death Discov..

[80] Prati F., Uliassi E., Bolognesi M.L. (2014). Two diseases, one approach: Multitarget drug discovery in Alzheimer’s and neglected tropical diseases. MedChemComm.

[81] Dorlo T.P., Balasegaram M., Beijnen J.H., de Vries P.J. (2012). Miltefosine: A review of its pharmacology and therapeutic efficacy in the treatment of leishmaniasis. J. Antimicrob. Chemother..

