# Supplementary material for: Naphthoquinone as a New Chemical Scaffold for Leishmanicidal Inhibitors of Leishmania GSK-3
Source: Biomedicines. 2022 May 14;10(5):1136. doi: 10.3390/biomedicines10051136 (PMC9139002; doi:10.3390/biomedicines10051136)
Supplement: Supplementary file 1 [file biomedicines-10-01136-s001.zip › biomedicines-1691278-supplementary.pdf]

Supplementary Material

Naphtoquinone as a new chemical scaffold for leishmanicidal inhibitors of *Leishmania* GSK-3

Victor Sebastián-Pérez,[1,&] Paula Martínez de Iturrate,[1,&] Montserrat Nácher-Vázquez,[1]

Luis Nóvoa,[1] Concepción Pérez,[2] Nuria E. Campillo,[2] Carmen Gil[1,*] and Luis Rivas[1,*]

[1]Centro de Investigaciones Biológicas (CIB-CSIC), Madrid, Spain

[2]Instituto de Química Médica (IQM-CSIC), Madrid, Spain

[&]Both authors contribute equally to this work

Index:

Page S2: Sequence alignment of the GSK-3 structures of *Leishmania major* (Q4QE15) and *Homo sapiens* (P49841).

Page S3: Chemical structures of the 24 compounds from the MBC library selected by virtual screening and their biological evaluation.

```
GSK3B_HUMAN  MSGRPRTTSFAESCKPVQQPSAFGSMKVSRDKDGSKVTTVVATPGQGPDRPQEVSYTDTK  60
Q4QE15_LEIMA ------------------------------------MSLNAAAAADERSRKEMDRFQVER  24
                                                      ::  .*  .:  .* :   :   :

GSK3B_HUMAN  VIGNGSFGVVYQAKLCDSGELVAIKKVLQDKRFKNRELQIMRKL---DHCNIVRLRYFFY  117
Q4QE15_LEIMA MAGQGTFGTVQLGKEKSTGMSVAIKKVIQDPRFRNRELQIMQDLAVLHHPNIVQLQSYFY  84
                  : *:*:**.*  .*  .:*  ******:** **:*******:.*   .* ***:*: :**

GSK3B_HUMAN  SSGEK-KDEVYLNLVLDYVPETVYRVARHYSRAKQTLPVIYVKLYMYQLFRSLAYIH--S  174
Q4QE15_LEIMA TLGERDRRDIYLNVVMEYVPDTLHRCCRNYYRRQVAPPPILIKVFLFQLIRSIGCLHLPS  144
               : **: : ::***:*::***:*::* .*:* * : : * * :*::::**:**:. :*  *

GSK3B_HUMAN  FGICHRDIKPQNLLLDPDTAVLKLCDFGSAKQLVRGEPNVSYICSRYYRAPELIFGATDY  234
Q4QE15_LEIMA VNVCHRDIKPHNVLVNEADGTLKLCDFGSAKKLSPSEPNVAYICSRYYRAPELIFGNQHY  204
               ..:*******:*:*::   ..**********:*  .****:***************  .*

GSK3B_HUMAN  TSSIDVWSAGCVLAELLLGQPIFPGDSGVDQLVEIIKVLGTPTREQIREMNPNYTEFKFP  294
Q4QE15_LEIMA TTAVDIWSVGCIFAEMMLGEPIFRGDNSAGQLHEIVRVLGCPSREVLRKLNPSHTDVDLY  264
               *:::*:**.**::**::**:*** **....** **::*** *:** :*::**.:*:..:

GSK3B_HUMAN  QIKAHPWTKVFRPRT---PPEAIALCSRLLEYTPTARLTPLEACAHSFFDELRDPNVKLP  351
Q4QE15_LEIMA NSKGIPWSNVFSDHSLKDAKEAYDLLSALLQYLPEERMKPYEALCHPYFDELHDPATKLP  324
               : *. **:.**  ::     **  * * **:* *  *:.* ** .* :****:*  .***

GSK3B_HUMAN  NGRDTPA-LFNFTTQELSSNPPLATILIPPHARIQAAASTPTNATAASDANTGDRGQTNN  410
Q4QE15_LEIMA NNKDLPEDLFRFLPNEIEVMSEAQKAKLVRK-----------------------------  355
                  *.:* *  **.*  .*:.      .  :  :

GSK3B_HUMAN     AASASASNST 420
Q4QE15_LEIMA    ----------355
```

Figure S1. Sequence alignment of the GSK-3 structures of *Leishmania major* (Q4QE15) and *Homo sapiens* (P49841). Some of the most important residues of the ATP binding site (Pocket 0) and substrate binding site are depicted in blue and green respectively; in red (Pocket 1), light blue (Pocket 4) and purple (Pocket 6 and 9).

Table S1. Enzymatic and biological characterization of the 24 compounds from the MBC library selected by virtual screening.

**Compound**	**Chemical formula**	**LdGSK-3s IC_{50} (µM)**	***L. infantum* promastigotes IC_{50} (µM)**	***L. pifanoi* axenic amastigotes IC_{50} (µM)**	**Murine peritoneal macrophages IC_{50} (µM)**	**Selectivity index (SI)**
**MBC-10**	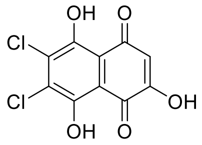	~10*	10.5 ± 1.2	11.2 ± 2.5	ND	ND
**MBC-49**	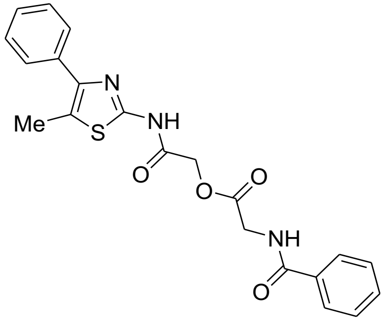	>10	>50	>50	-	-
**MBC-61**	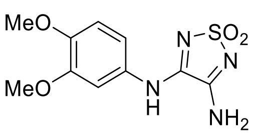	>10	>50	>50	-	-
**MBC-85**	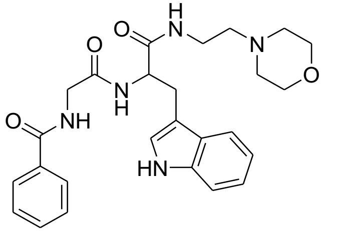	>10	>50	>50	-	-

* MBC-10 at 10 µM causes 45.7 ± 7.6 % inhibition of LdGSK-3s activity.

Compound	Chemical formula	LdGSK-3s IC$_{50}$ (µM)	*L. infantum* promastigotes IC$_{50}$ (µM)	*L. pifanoi* axenic amastigotes IC$_{50}$ (µM)	Murine peritoneal macrophages IC$_{50}$ (µM)	Selectivity index (SI)
**MBC-11**	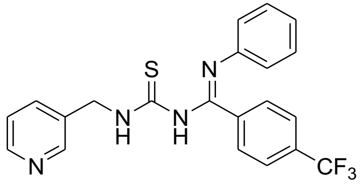	>10	>25	>25	-	-
**MBC-188**	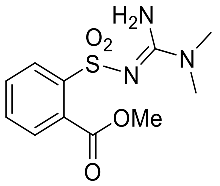	>10	>50	>50	-	-
**MBC-189**	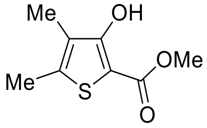	>10	>50	>50	-	-
**MBC-190**	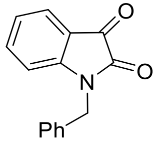	>10	>50	>50	-	-
**MBC-191**	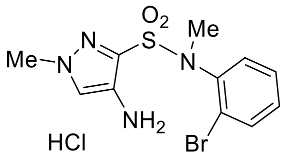	>10	>50	>50	-	-

Compound	Chemical formula	LdGSK-3s IC_{50} (µM)	*L. infantum* promastigotes IC_{50} (µM)	*L. pifanoi* axenic amastigotes IC_{50} (µM)	Murine peritoneal macrophages IC_{50} (µM)	Selectivity index (SI)
**MBC-192**	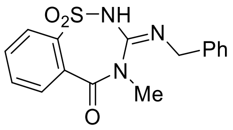	>10	>50	>50	-	-
**MBC-193**	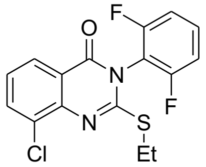	>10	>50	>50	-	-
**MBC-194**	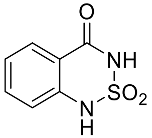	>10	>50	>50	-	-
**MBC-195**	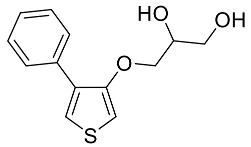	>10	>50	>50	-	-
**MBC-196**	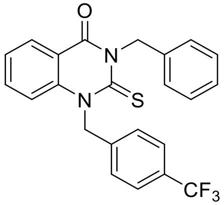	>10	>25	>50	-	-

Compound	Chemical formula	LdGSK-3s IC_{50} (µM)	*L. infantum* promastigotes IC_{50} (µM)	*L. pifanoi* axenic amastigotes IC_{50} (µM)	Murine peritoneal macrophages IC_{50} (µM)	Selectivity index (SI)
**MBC-197**	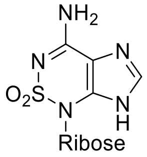	>10	>50	>50	-	-
**MBC-198**	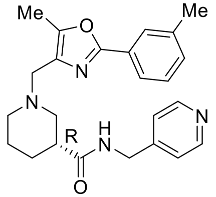	>10	>50	>50	-	-
**MBC-199**	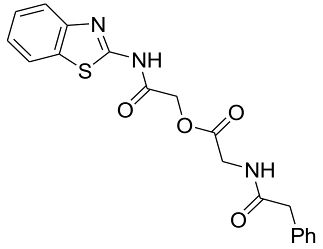	>10	>50	>50	-	-
**MBC-200**	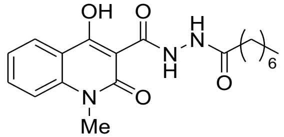	>10	>50	20.8 ± 0.0	>25	>1.2
**MBC-201**	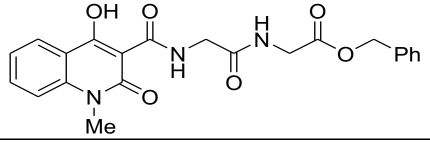	>10	>50	>50	-	-

Compound	Chemical formula	LdGSK-3s IC_{50} (µM)	*L. infantum* promastigotes IC_{50} (µM)	*L. pifanoi* axenic amastigotes IC_{50} (µM)	Murine peritoneal macrophages IC_{50} (µM)	Selectivity index (SI)
**MBC-202**	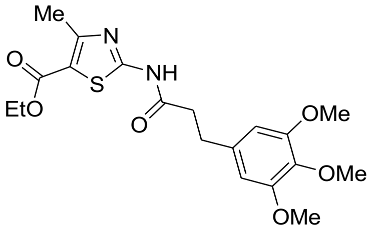	>10	>50	>50	-	-
**MBC-203**	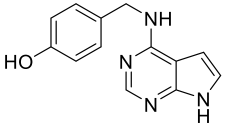	>10	>50	>50	-	-
**MBC-204**	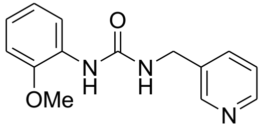	>10	>50	>50	-	-
**MBC-205**	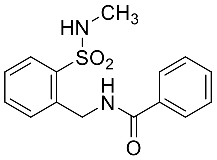	>10	>50	>50	-	-
**MBC-206**	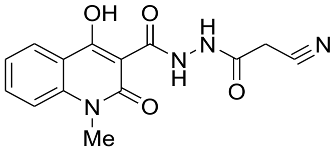	>10	>50	>50	-	-
